# Insulator function and topological domain border strength scale with architectural protein occupancy

**DOI:** 10.1186/gb-2014-15-5-r82

**Published:** 2014-06-30

**Authors:** Kevin Van Bortle, Michael H Nichols, Li Li, Chin-Tong Ong, Naomi Takenaka, Zhaohui S Qin, Victor G Corces

**Affiliations:** 1 Department of Biology, Emory University, 1510 Clifton Road NE, Atlanta, GA 30322, USA; 2 Department of Biostatistics and Bioinformatics, Emory University, Atlanta, GA 30322, USA

## Abstract

**Background:**

Chromosome conformation capture studies suggest that eukaryotic genomes are organized into structures called topologically associating domains. The borders of these domains are highly enriched for architectural proteins with characterized roles in insulator function. However, a majority of architectural protein binding sites localize within topological domains, suggesting sites associated with domain borders represent a functionally different subclass of these regulatory elements. How topologically associating domains are established and what differentiates border-associated from non-border architectural protein binding sites remain unanswered questions.

**Results:**

By mapping the genome-wide target sites for several *Drosophila* architectural proteins, including previously uncharacterized profiles for TFIIIC and SMC-containing condensin complexes, we uncover an extensive pattern of colocalization in which architectural proteins establish dense clusters at the borders of topological domains. Reporter-based enhancer-blocking insulator activity as well as endogenous domain border strength scale with the occupancy level of architectural protein binding sites, suggesting co-binding by architectural proteins underlies the functional potential of these loci. Analyses in mouse and human stem cells suggest that clustering of architectural proteins is a general feature of genome organization, and conserved architectural protein binding sites may underlie the tissue-invariant nature of topologically associating domains observed in mammals.

**Conclusions:**

We identify a spectrum of architectural protein occupancy that scales with the topological structure of chromosomes and the regulatory potential of these elements. Whereas high occupancy architectural protein binding sites associate with robust partitioning of topologically associating domains and robust insulator function, low occupancy sites appear reserved for gene-specific regulation within topological domains.

## Background

The recently developed high-throughput chromosome conformation capture (3C)-based molecular techniques have propelled our understanding of three-dimensional chromosome organization to new heights. In particular, the organization of eukaryotic genomes into discrete physical domains can now be defined by surveying genome-wide pairwise interaction frequencies. A series of such analyses in *Drosophila*, mice, and humans have provided insights into the hierarchical organization of interphase chromosomes on different length scales, and raise additional questions on the mechanisms governing three-dimensional genome organization [1-9]. During interphase, genomes are partitioned into sub-megabase length topologically associating domains (TADs), which are further organized into multi-megabase sized structures called compartments, whose distribution often reflects cell type-specific expression patterns [10]. In contrast, TAD structure is generally consistent between diverse cell types [3], suggesting the sub-megabase scale arrangement of chromosomes may represent a conserved, bottom-up pattern of chromatin organization and genome function. Thus, understanding how TADs are established and maintained between cell types remains an important question.

Integration of long-range interaction frequencies and domain organization with genomic annotations along the linear genome has revealed a strong relationship between TAD borders and proteins associated with insulator function. For example, CTCF (CCCTC-binding factor) as well as tRNA genes (tDNAs), recently shown by transgene protection assays to possess classical insulator activity in humans [11,12], are significantly enriched in regions separating topological domains [3]. Nevertheless, 85% of CTCF binding sites localize within TADs rather than at their borders, suggesting most CTCF sites are unrelated to the formation of borders that separate TADs. Meanwhile, multiple studies suggest that many insulator elements are not capable of enhancer-blocking or chromatin barrier activity at all [13-15], and may instead be reserved for other activities such as gene repression, activation, or enhancer-promoter interactions [16-18]. The seemingly contradictory activities of insulators and the dichotomy of border-associated versus non-border target sites suggest that the very use of the name ‘insulator’ is, in most cases, erroneous. To avoid further sustaining this confusion, we hereafter refer to proteins associated with insulator function as architectural proteins, and refer to insulators only in the context of elements capable of enhancer-blocking activity.

To date, several architectural proteins have been identified in *Drosophila melanogaster*, including the *Drosophila* homolog of CTCF (dCTCF), Suppressor of hairy-wing (Su(Hw)), GAGA factor (GAF), and the scs and scs’ boundary proteins Boundary element associated factor of 32 kDa (BEAF-32) and Zeste white 5 (Zw5) [19]. Phylogenetic analyses in *Drosophila* suggest, however, that all but dCTCF and Su(Hw) were successively gained during arthropod evolution [20], and that additional and perhaps unexplored architectural proteins may supplement the highly conserved CTCF protein in vertebrates. Supporting evidence for this possibility comes from recent genome-wide mapping studies of the multisubunit RNA polymerase (Pol) III transcription factor TFIIIC, which is essential for the inherent insulator activity of tRNA genes in yeast [21]. In mammals, TFIIIC often binds to Pol III-independent regions, called extra TFIIIC (ETC) loci, in close proximity to CTCF [22,23]. TFIIIC binding sites also associate with the cohesin complex in mammals [23], and can also underlie condensin loading onto chromosomes in *Saccharomyces cerevisiae*[24], strongly suggestive of a role in chromatin organization. Understanding the function of TFIIIC and its relationship to other architectural proteins may therefore shed light on the mechanisms by which these proteins contribute to the three-dimensional organization of the genome in the nucleus.

Here we present the first genome-wide characterization of TFIIIC in *D. melanogaster* and find that this protein localizes to sites combinatorially bound by several *Drosophila* architectural proteins. These high occupancy architectural protein binding sites (APBSs) localize to the borders of TADs, are enriched for both the cohesin and condensin complexes, and represent highly accessible regions of chromatin that are stable throughout *Drosophila* development, consistent with the tissue-invariant nature of TADs observed in mammals. The relative occupancy of architectural proteins at APBSs scales with the strength of TAD borders, as well as the capacity of these elements to function as enhancer-blocking insulators in transgenic reporter assays, suggesting the composition of these regulatory elements underlies a spectrum of regulatory potential. Finally, we uncover a similar relationship between TFIIIC, CTCF, cohesin, condensin and TADs in mice and humans, suggesting a conserved role for clustered architectural proteins in sub-megabase scale chromatin domain organization.

## Results

### Characterization and genome-wide mapping of TFIIIC in *D. melanogaster*

TFIIIC targets sequence-specific gene-internal A box and B box promoter elements present in a subset of Pol III-transcribed genes [25], where it then recruits the transcription factor complex TFIIIB. Biochemical and molecular characterization of TFIIIC has revealed evolutionary changes in protein structure and protein-protein interactions between yeast and humans, yet the subunit composition is generally conserved [26]. In *D. melanogaster*, the protein-coding gene *CG7099* (Flybase FBgn0032517) is predicted to encode a B box binding subunit of TFIIIC based on protein sequence homology. Immunoblot and immunofluorescence localization of CG7099, which we now refer to as dTFIIIC220, confirms an antigen-specific protein at the predicted molecular weight (approximately 220 kDa), which localizes to numerous binding sites in polytene chromosomes throughout the *Drosophila* genome (Figure S1a-f in Additional file 1).

**Figure 1:**
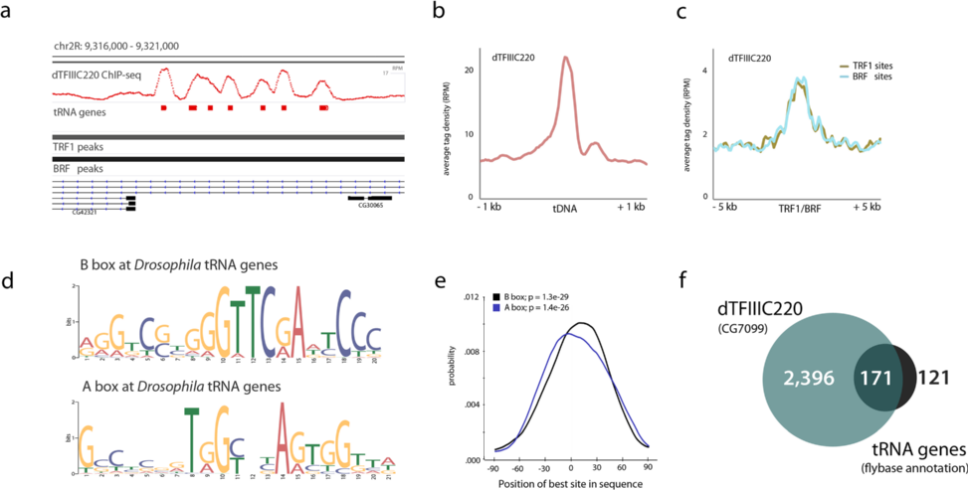
**Genome-wide mapping of dTFIIIC220 in *D. melanogaster*. (a)** Example ChIP-seq profile shown for dTFIIIC220 (red) over a tRNA cluster on *Drosophila* chromosome 2R, co-bound by TFIIIB subunits TRF1 and BRF. **(b,c)** Tag density enrichment profiles for dTFIIIC220 over all annotated tDNAs (B) and over sites previously identified as bound by TFIIIB complex subunits TRF1 and BRF (C) confirms the expected genome-wide localization patterns for *Drosophila* (overlap significance *P* < 0.00001, permutation test). RPM, reads per million. **(d)** Consensus sequences identified *de novo* by MEME-ChIP reveals evolutionarily conserved *Drosophila* B box and A box elements present in dTFIIIC220-bound tRNA genes. **(e)** Central motif enrichment (CentriMo) plot for B box and A box sequences with respect to dTFIIIC220 ChIP-seq peaks at tRNA genes. **(f)** Overlap between dTFIIIC220 peaks, independently identified in two biological replicates at a false discovery rate of 5%, with annotated tRNA genes obtained from Flybase (*P* < 0.00001, permutation test). Non-overlapping sites indicate thousands of ETC loci in *D. melanogaster*, of which 348 contain the B box binding motif (14.5%, *P* < 0.00001, permutation test).

We performed ChIP-seq against dTFIIIC220 in Kc167 cells as recently carried out for several DNA-binding factors [15,27]. Genome-wide analysis confirms the localization of dTFIIIC220 to tRNA genes and sites associated with the TFIIIB complex as expected (Figure 1a-c), and MEME-ChIP and CentriMo consensus sequence analysis further demonstrates central motif enrichment for both the *Drosophila* A box and B box elements in our ChIP-seq experiments (Figure 1d,e) [28,29]. dTFIIIC220 binding sites determined by the commonly used MACS peak calling algorithm [30] are present at a majority of annotated tRNA genes obtained from Flybase (Figure 1f) [31], and dTFIIIC220 reads are significantly enriched over all annotated tRNA genes and TFIIIB subunit (TRF1 and BRF) binding sites (Figure 1b,c). In addition to tRNA genes, we identify numerous ETC loci (Figure 1f) independent of tRNA gene structure or TFIIIB localization, suggesting *Drosophila* TFIIIC may also function at sites independent of Pol III transcription, as is the case for TFIIIC in mammals [22,23].

### Relationship to SMC-containing cohesin and condensin complexes

Mammalian CTCF recruits and depends on cohesin for functional insulator activity [32-34], and original tDNA-based insulator studies in *S. cerevisiae* observed an analogous dependency on SMC proteins [21]. TFIIIC-bound B box elements can also constitute functional loading sites for the condensin complex in *S. cerevisiae*[24], and multiple studies have described a role for condensin in the organization of dispersed Pol III genes in *Schizosaccharomyces pombe*[35,36], suggesting TFIIIC activity is tightly associated with SMC complexes. We therefore mapped the genomic binding profile for cohesin and the paralogous condensin complexes via complex specific α-kleisin subunits Rad21 (cohesin), Barren (condensin I), and CAP-H2 (condensin II) to better understand their possible relationship to dTFIIIC220 in *D. melanogaster*.

**Figure 2:**
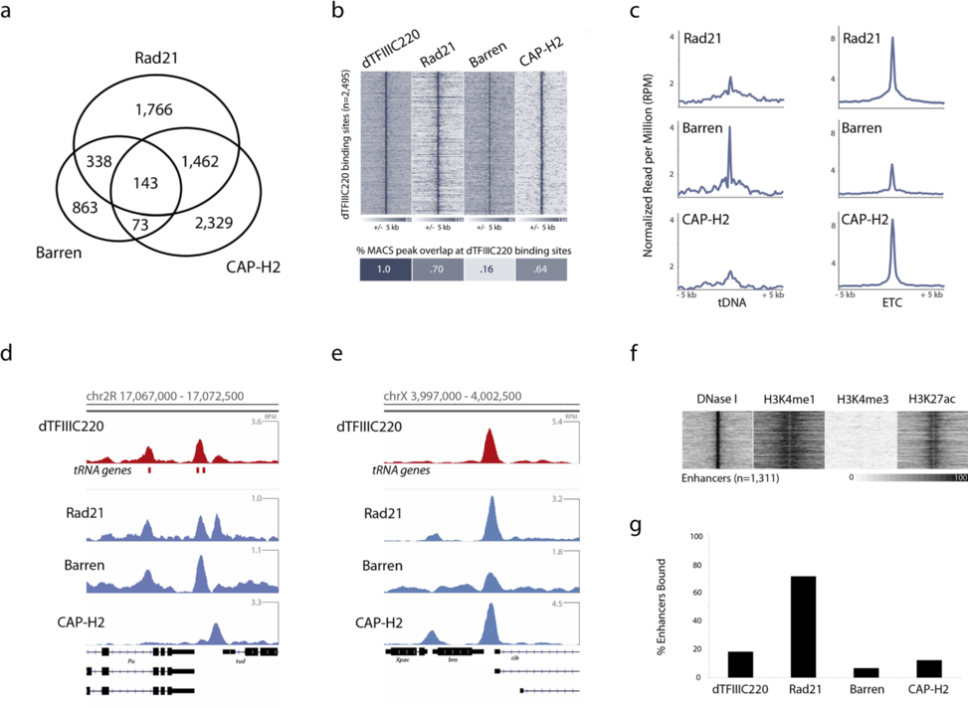
**SMC-containing cohesin and condensin complexes localize to a subset of tDNAs and ETC loci. (a)** Number of overlapping peaks identified by ChIP-seq against α-kleisen subunits Rad21 (cohesin), Barren (condensin I), and CAP-H2 (condensin II) in Kc167 cells (*P* < 0.00001 for overlap between Rad21 with CAP-H2 or Barren, permutation test). **(b)** Heatmap representation of ChIP-seq read intensities of SMC-containing complexes and TFIIIC subunit dTFIIIC220, anchored across all dTFIIIC220 peaks (top), plus or minus 5 kb. Heatmap representation (bottom) of overlap frequencies between dTFIIIC220 peaks and those of SMC-containing complexes (overlap significance for dTFIIIC220 with each factor *P* < 0.00001, permutation test). **(c)** Read intensity plots for Rad21, Barren, and CAP-H2 at TFIIIC-bound tDNAs (left) and ETC loci (right) plus or minus 5 kb. Tag density is represented as rank-order normalized reads per million (RPM) across all three ChIP-seq experiments. **(d,e)** Example genomics viewer profiles of overlapping dTFIIIC220 sites at tRNA genes and ETC loci. **(f)** Heatmap representation shown for DNase-seq and ChIP-seq read intensities at 1,311 active enhancers previously defined by STARR-seq, and marked by active enhancer characteristics in the Kc167 cell line, including DNase I hypersensitivity, H3K4me1 and H3K27ac. **(g)** Percentage of enhancers bound by dTFIIIC220 and SMC-containing complexes.

Analysis of the cohesin and condensin binding profiles in *Drosophila* Kc167 cells reveals substantial overlap between the three SMC-containing complexes (Figure 2a). Further comparison with dTFIIIC220 indicates strong co-localization at ETC loci, particularly for the cohesin and condensin II complexes (Figure 2b), suggesting that association with cohesin and condensin at TFIIIC sites is conserved in *Drosophila*. Additionally, we find that whereas condensin I is most pronounced at tRNA genes (Figure 2c,d), consistent with recent condensin mapping studies in vertebrate chicken DT40 cells [37], both cohesin and condensin II are present at higher levels at ETC loci (Figure 2c,e). This distinction in cohesin and condensin association suggests a unique specialization of SMC complex recruitment to TFIIIC binding sites, possibly underlying differences in co-factor colocalization patterns and function. We therefore next sought to characterize ETC loci and their potential role in genome function.

Previous genome-wide mapping studies in *Drosophila* and mammalian cells have shown that cohesin often localizes to highly occupied *cis*-regulatory modules that may function as developmental or cell type-specific enhancers [38-41], and both cohesin and condensin II localize to super-enhancers reported to be involved in controlling mammalian cell identity [42]. We thus compared the profiles for TFIIIC, cohesin, and condensin complexes with 1,311 previously reported enhancers characterized by DNase I hypersensitivity (DHS) and enhancer hallmarks H3K4me1 and H3K27ac in Kc167 cells (Figure 2f) [43]. A large majority of enhancers are bound by the cohesin complex (Figure 2g) and, unlike Pol II, cohesin is more significantly enriched at individual enhancers than transcription start sites (Figure S2a in Additional file 2). However, very few enhancers are bound by dTFIIIC220, and fewer yet associate with the condensin complexes (Figure 2g), suggesting sites co-bound by TFIIIC and SMC complexes generally do not represent active enhancers.

### TFIIIC clusters with CTCF and other *Drosophila* architectural proteins

**Figure 3:**
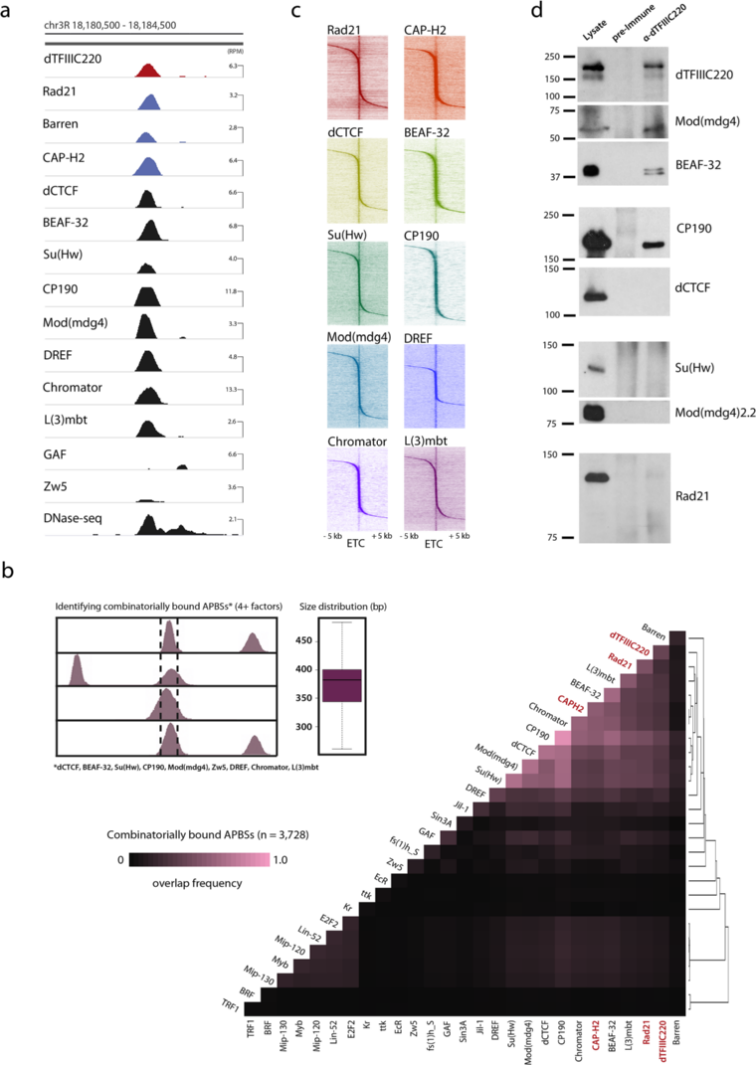
***Drosophila *TFIIIC clusters with CTCF at sites combinatorially bound by architectural proteins, cohesin, and condensin II. (a)** Example genomics viewer profile of a combinatorially bound APBS, co-bound by dTFIIIC220, SMC-containing cohesin and condensin complexes, dCTCF, BEAF-32, Su(Hw), CP190, Mod(mdg4), DREF, Chromator, L(3)mbt, and marked by strong DHS. **(b)** Heatmap representation of co-factor co-localization at 3,728 genomic loci combinatorially bound by architectural proteins. Overlap frequency is the fraction of combinatorially bound loci bound by each individual factor. Inset: sites were identified as genomic fragments having four or more proteins in Kc167 cells using MACS called summits ±200 bp for factors dCTCF, BEAF-32, Su(Hw), CP190, Mod(mdg4), Zw5, DREF, Chromator, and L(3)mbt, and mapped independently of TFIIIC and SMC complexes; size distribution (bp) of combinatorially bound loci. *P* < 0.00001 for overlap between combinatorially bound loci with dTFIIIC220, Rad21, and CAP-H2, permutation test. Overlap frequency matrix hierarchically clustered (absolute centered, single linkage). **(c)** Heatmaps depict ChIP-seq tag densities for each *Drosophila* architectural protein as a function of distance, ±5 kb, from ETC loci. **(d)** Western blot analysis of control preimmune and α-dTFIIIC220 immunoaffinity purifications detect interactions between dTFIIIC220 and CP190, Mod(mdg4), and BEAF-32.

Visual inspection of dTFIIIC220 ChIP-seq data instead suggests that TFIIIC target regions coincide with sites marked by previously characterized architectural proteins. In particular, dTFIIIC220 binding sites often localize to regions combinatorially bound by several factors shown to associate with insulator activity (Figure 3a). These high occupancy APBSs also correlate with SMC-containing cohesin and condensin complexes, consistent with the strong correlation observed with TFIIIC. This finding is surprising, however, as previous ChIP-chip studies mapping an ancillary cohesin subunit, Scc3, observed a relatively weak overlap with dCTCF [44], which, like BEAF-32 and Su(Hw), recruits BTB-containing proteins CP190 and Mod(mdg4) essential for insulator activity [45-47]. These original observations have led to speculation that *Drosophila* CTCF functions through a unique mechanism compared to its mammalian counterpart, yet our genome-wide high-resolution profile of Rad21 suggests a more extensive co-localization between CTCF and cohesin in *Drosophila*. For example, nearly half of all high confidence CTCF binding sites identified in three biological replicates correlate with Rad21, similar to numbers originally identified in vertebrate HeLa cells [33], and Rad21 chromatin immunoprecipitation (ChIP) enrichment is significantly greater at APBSs than at independent loci (Figure S2a-d in Additional file 2). Furthermore, depletion of dCTCF by RNA interference (RNAi) in Kc167 cells disrupts Rad21 localization specifically to dCTCF binding sites (Figure S2e-g in Additional file 2), suggesting recruitment of cohesin is conserved from *Drosophila* to mammals.

Genome-wide, dTFIIIC220, Rad21, and CAP-H2 strongly associate with combinatorially bound APBSs, independently determined to contain four or more previously characterized architectural proteins. Hierarchical clustering of overlap frequencies observed between TFIIIC, SMC complexes and defined transcription factor binding sites in Kc167 cells illustrates this relationship, wherein dTFIIIC220, Rad21, and CAP-H2 cluster with architectural proteins at these loci (Figure 3c). For example, out of 3,728 combinatorially bound APBSs, 1,489 (40%), 2,124 (57%), and 1,830 (49%) are associated with dTFIIIC220, CAP-H2, or Rad21, respectively (*P* < 0.00001, permutation test). We observe a comparatively weak overlap with transcription factor binding sites identified in Kc167 cells (Figure 3c), suggesting colocalization patterns observed for these architectural proteins are different from transcription factor hotspots. However, the enrichment of Rad21 at high occupancy APBSs is intriguing, as cohesin was recently shown to maintain high occupancy transcription factor clusters in mammals [39,40].

In order to determine whether TFIIIC might directly interact with *Drosophila* architectural proteins, dTFIIIC220-associated complexes were isolated by immunoaffinity purification (Figure 3d). Western blot analysis of control preimmune and α-dTFIIIC220 immunoaffinity purifications suggests that TFIIIC associates with both CP190 and Mod(mdg4), as is the case for other *Drosophila* architectural proteins [15,45-47]. Although a comparatively weak interaction is detected with BEAF-32, dTFIIIC220 does not appear to directly associate with dCTCF or Su(Hw), and we could not detect an interaction with Rad21, suggesting the dTFIIIC220 subunit may not directly recruit cohesin via α-kleisin subunit Rad21. Nevertheless, interactions with CP190 and Mod(mdg4) extend a common theme observed for proteins associated with insulator function in *D. melanogaster* to TFIIIC, suggesting BTB-containing proteins may also represent a unifying mechanism for both long-range interactions as well as co-occupancy at these sites.

### Clustering of architectural proteins scales with TAD border strength

Analyses of TADs in *D. melanogaster* consistently demonstrate that architectural proteins are highly enriched at boundary regions flanked by two adjacent domains [1,2]. We therefore sought to define high occupancy APBSs by cross-analyzing ChIP-seq data against binding data for dTFIIIC220, Rad21, CAP-H2, dCTCF, BEAF-32, Su(Hw), CP190, Mod(mdg4), the transcription factor DREF [48], the chromo-domain protein Chromator, previously shown to colocalize and co-immunoprecipitate with BEAF-32 [49], and the tumor suppressor L(3)mbt protein, recently shown to localize specifically to *Drosophila* APBSs [50] (list provided in Additional file 3). We further classified overlapping binding sites based on the number of overlapping proteins into sites with high, medium, or low occupancy (Figure S3a in Additional file 4). High occupancy APBSs correlate with regions associated with strong DHS sites [51], and associate with increasing DHS intensity as measured by DNase-seq in Kc167 cells (Figure S3b in Additional file 4), suggesting APBSs represent open chromatin regions whose accessibility increases with increasing cofactor occupancy. Analysis of the location of APBSs with respect to gene structure indicates that high occupancy sites are more likely to reside in regions that are upstream and proximal to transcription start sites, analogous to colocalization patterns recently observed for overlapping mammalian factors [39]. Nevertheless, DHS centers on APBSs and is independent of the proximity of these regions with gene promoters.

**Figure 4:**
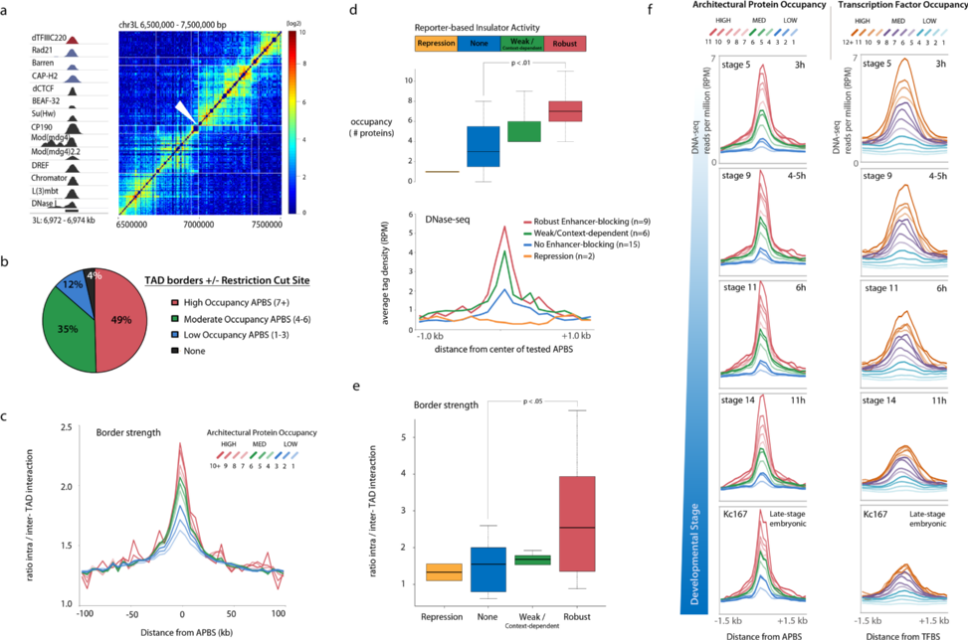
**High occupancy APBSs delineate TADs and associate with robust enhancer-blocking activity. (a)** Heatmap representing Hi-C interaction frequencies at single fragment resolution for a 1 Mb region across *Drosophila* chromosome 3 L in Kc167 cells. White lines demarcate previously defined TAD boundaries [1]. A high occupancy APBS (left) is present at a single fragment topological domain border strongly separating two TADs (white arrowhead). Colorbar represents (log2) interaction frequencies observed between restriction fragments, ranging from low (blue) to high (red). **(b)** Percentage of TADs defined in Kc167 cells delineated by a high, medium, or low occupancy APBSs ± one restriction cut site (TAD borders n = 1,110, high occupancy APBSs n = 1,638, *P* < 0.00001, permutation test). **(c)** Topological border strength defined by the ratio of intra- versus inter-TAD interaction frequencies scales with the occupancy (number of bound proteins) at APBSs. **(d)** Architectural protein occupancy and DNase I hypersensitivity at DNA fragments previously tested for enhancer-blocking activity in transgenic reporter assays [13,51,52]. Sequences shown to possess robust activity (red) correlate with both the highest occupancy and DNase I activity, whereas sites incapable of insulator activity are marked by low occupancy (*P* < 0.01, Wilcoxon rank sum test, two-sided). RPM, reads per million. **(e)** Quantification of topological domain border strengths at sequences tested for insulator function within their endogenous context. Robust insulator sequences are characterized by significantly greater topological border strength than non-enhancer-blocking sequences (*P* < 0.05, Wilcoxon rank sum test, two-sided). **(f)** Tag density plots of rank-order normalized DNase-seq profiles throughout embryonic stages of development at APBSs [53], and at transcription factor binding sites shown to function as developmental enhancers during early embryogenesis. The progressive loss of DNase accessibility at highly bound transcription factor binding sites (right) contrasts with the combinatorially bound APBSs (left), which are marked by strong DNase I hypersensitivity throughout each stage of development.

Comparison of protein occupancy with respect to TAD localization further reveals a significant enrichment for high occupancy APBSs near TAD borders previously identified by high-throughput chromosome conformation capture [1]. For example, a strong domain border can be observed at 7 × 10^6^ bp on *Drosophila* chromosome 3L in the form of two TADs defined by high intra-domain interaction frequencies and low inter-domain interaction frequencies (Figure 4a). The single fragment resolution TAD boundary identified corresponds to a region containing a high occupancy APBS bound by all queried proteins, including dTFIIIC220, suggesting strong chromatin domain separation may be collectively orchestrated by several architectural proteins. Genome-wide, protein occupancy is a strong predictor of TAD border localization, wherein 49% of TAD boundaries defined in Kc167 cells [1] are delineated within one restriction cut site by a high occupancy APBS, 35% by a medium occupancy APBS, and 12% by a low occupancy APBS (Figure 4b). We find similar enrichment profiles at TAD borders defined by Hi-C in embryos [2], and that localization to domain borders is independent of gene structure (Figure S3e,f in Additional file 4; Additional file 5).

TADs are defined by the compartmentalization of interaction frequencies, yet they also show varying degrees of compartmentalization. In other words, the borders that separate TADs appear to vary in terms of strength. We therefore quantified the degree of domain separation, or ‘border strength’, by measuring the ratio of intra- versus inter-TAD interaction frequencies (Materials and methods). Comparison of APBSs with border strength reveals a striking relationship, wherein chromatin domain separation scales incrementally with architectural protein occupancy (Figure 4c), providing strong evidence that combinatorial binding of these factors underlies a spectrum of functional capacity. In addition to differences in domain border strength, TADs also vary widely in size, ranging from only a few to several hundred kilobases in length. Visualization of pairwise interaction frequencies on a megabase scale illustrates this heterogeneity, which scales with the density and occupancy of APBSs (Figure S5a-c in Additional file 6). Whereas dense regions of high occupancy APBSs associate with very small TADs (median size approximately 55 kb), genomic regions characterized by low densities of clustered architectural proteins are comparatively much larger (median size 145 to 180 kb), consistent with a role for high occupancy APBSs in chromatin domain separation.

### High occupancy APBSs associate with robust enhancer blocking activity

The role and function of insulator elements in genome biology has remained difficult to describe, despite extensive characterization and analyses. Though first defined by their ability to insulate genes from position effects and to prevent enhancer-promoter communication in transgenic reporter assays, many endogenous APBSs appear to lack these defining characteristics [13], suggesting they do not represent ‘insulators’ in the classical sense. In agreement with this, recent work in mammals suggests that many CTCF sites fail to interfere with enhancer-promoter interactions and that their role may be to facilitate interactions between these regulatory sequences instead [16,17]. We therefore analyzed the relative occupancy level of architectural proteins in DNA fragments previously tested for enhancer-blocking activity using reporter assays, wherein specific regions of the genome were shown to be capable of robust or context-dependent enhancer blocking, incapable of enhancer-blocking activity, or act instead as transcriptional repressors [13,52].Insulator sequences capable of robust enhancer-blocking activity indeed correlate with high occupancy APBSs, with an average occupancy of 7.1 factors (Figure 4d). We find an intermediate level of protein occupancy at context-dependent insulators (5.2 factors), and comparatively low occupancy at fragments that did not possess enhancer-blocking activity (3.5 factors). The gradient of insulator activity correlates with DHS, consistent with the observed occupancy level and suggesting that robust enhancer-blocking insulators represent chromatin bound by several architectural proteins (Figure 4d).

Analysis of these sequences with respect to TAD border strength within their endogenous contexts further confirms that reporter-based assays reflect the functional capacity of these elements *in vivo*. For example, robust enhancer-blocking sequences correspond with genomic regions associated with strong TAD border strength, whereas non- or weak enhancer-blocking elements associate with weak border strength (Figure 4e). These data suggest that highly occupied APBSs enriched at the borders of TADs represent strong insulators involved in chromatin domain organization, whereas sites bound individually or by few architectural proteins reside within TADs and may be reserved for specific regulation of genes.

### High occupancy APBSs are characterized by DNase I hypersensitivity throughout *Drosophila* development

Genome-wide chromosome conformation capture studies provide evidence that a majority of topological domains are tissue invariant [3], suggesting sub-megabase scale domain structure may represent a common framework for higher order organizational dynamics. If clustered architectural proteins function to establish or maintain TADs, then high occupancy APBSs too must be largely tissue invariant and present throughout *Drosophila* development. We therefore compared APBSs defined in *Drosophila* Kc167 cells with DHS profiles captured throughout stages of embryogenesis as a proxy for both chromatin accessibility and protein occupancy [51,53]. DNase-seq profiles were rank-order normalized (Materials and methods) across five embryonic stages, including the late-stage Kc167 cell line, and plotted with respect to protein occupancy at APBSs (Figure 4f).

High occupancy APBSs show a remarkably consistent pattern of DHS intensity, even at the earliest embryonic stages of development tested, just 3 hours post-fertilization (Figure 4f), suggesting they are indeed stably occupied. Importantly, DHS is consistent across both promoter- and non-promoter-associated clusters (Additional file 5), supporting the use of chromatin accessibility as a measure of protein occupancy. The consistently open chromatin status at high occupancy APBSs starkly contrasts with the DNase I profiles of previously characterized transcription factor HOT regions, which instead gradually lose DNase accessibility across embryonic stages (Figure 4f). The loss of DHS intensity at sites co-bound by several early transcription factors is consistent with data suggesting HOT regions function as spatiotemporal specific developmental enhancers during early embryogenesis [38]. These findings suggest that, unlike HOT sites, clustered APBSs remain highly occupied throughout *Drosophila* development, and thus denote stable hubs for architectural protein association that may underlie the conserved topological domain structure observed across diverse cell types.

### Mammalian TFIIIC and CTCF cluster at TAD borders

The observation that architectural proteins form large clusters and scale with the strength of TAD borders is made possible by the large repertoire of factors characterized to be essential for insulator function and mapped by ChIP-seq in *Drosophila*. This phenomenon has not been studied in mammals, however, due to our limited understanding of what factors, besides CTCF, are capable of insulator function in vertebrates. Recent discovery that tRNA genes possess insulator activity in humans [11] suggest that TFIIIC may be responsible for this function, and raise the possibility that clustering of architectural proteins may have functional significance in mammals as well. For example, ETC loci often localize near CTCF sites in both human cells and mouse embryonic stem cells (ESCs), and similarly associate with the cohesin complex as well [22,23]. We therefore asked whether TFIIIC and CTCF cluster together at topological domain borders by analyzing recent Hi-C data from mouse and human ESCs (mESCs and hESCs) and IMR90 fibroblasts [3].

**Figure 5:**
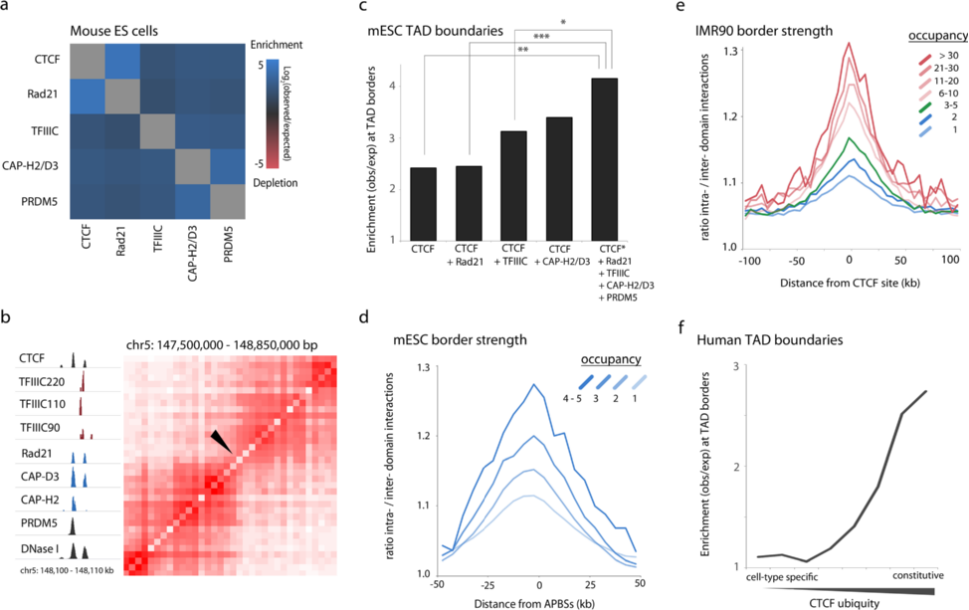
**Clustering of architectural proteins is a conserved feature of genome organization. (a)** Heatmap depicting overlap enrichment between architectural proteins mapped by ChIP-seq in mESCs. Red to blue squares represent depletion (red) or enrichment (blue), determined as the log_2_ (observed/expected) frequency of overlap when compared to randomized, simulated data. **(b)** Example genomics viewer profile (left) of a high occupancy APBSs in mESCs, bound by CTCF, TFIIIC (-220, -110, and -90), Rad21, condensin II (CAP-D3 and CAP-H2), and PRDM5, and marked by strong DHS. Hi-C interaction matrix (right) illustrates the corresponding TAD separation observed *in vivo* (TAD boundary defined by black arrowhead). **(c)** Sites combinatorially bound by CTCF and other factors (CTCF plus three or more proteins) are significantly enriched at TAD borders in mESCs. *P* values (*P < 0.05, ** *P* < 0.01, *** *P* < 0.001) were calculated using permutation tests. **(d)** Relationship between protein occupancy, defined by the presence of CTCF, Rad21, PRDM5, TFIIIC (any or all subunits -220, -110, -90) and condensin II (CAP-H2 and/or CAP-D3), and topological domain border strength in mESCs. **(e)** Parallel analysis of topological domain border strength in human IMR90 fibroblasts as a function of protein occupancy at CTCF binding sites. Co-binding determined by cross-comparison of ChIP-seq datasets for transcription factors and DNA binding proteins in human K562 cells. **(f)** Relationship between cell-type specificity of CTCF binding sites and localization to TAD borders. CTCF ubiquity determined by cross-comparison of 62 CTCF ChIP-seq datasets across 31 human cell lines. The x-axis represents CTCF sites grouped into eight bins (approximately 15,000 sites each) of increasing ubiquity ranging from cell type-specific to constitutive. For a list of human cell lines, ubiquity scores and exact number of CTCF binding sites in each bin, see Materials and methods and Additional file 8.

Comparison of ChIP-seq data mapping CTCF, cohesin, and three subunits of TFIIIC (TFIIIC220, -110, and -90) in mESCs indicates strong overlap among these proteins (Figure 5a). Furthermore, we find enrichment for condensin II subunits CAP-H2 and CAP-D3, consistent with colocalization patterns in *Drosophila*, as well as PRDM5, a SET domain protein recently shown to interact and co-occupy genomic loci with CTCF, TFIIIC, and cohesin [54]. Binding of these five distinct factors was therefore used as a proxy for occupancy at CTCF sites analogous to APBSs in *Drosophila*. Analysis of CTCF occupancy with respect to TAD borders in mESCs again demonstrates a strong correlation between architectural protein clustering and chromatin organization. For example, a strong TAD border mapped to chromosome 5 in mESCs corresponds to a region bound by CTCF, TFIIIC (-220, -110, -90), Rad21, Condensin II (CAP-H2 and CAP-D3) and PRDM5, and marked by strong DHS (Figure 5b). Occupancy at CTCF sites is a strong predictor of both TAD border localization (Figure 5c) and TAD border strength (Figure 5d) as observed for APBSs in *D. melanogaster*, suggesting that clustering of architectural proteins is a general feature of genome organization conserved between *Drosophila* and mammals.

Genome-wide mapping of human architectural proteins associated with insulator activity has, to date, been limited to CTCF, TFIIIC-110, and cohesin. Nevertheless, we find that sites occupied by all three factors are significantly enriched within TAD borders mapped in human ESCs and IMR90 fibroblasts, particularly at borders shown to be conserved between these two cell types (Figure S6a in Additional file 7). To gain better insight into the occupancy of CTCF binding sites, we took advantage of recent large-scale mapping studies in which more than 100 transcription factors and DNA binding proteins were mapped by ChIP-seq in the human K562 cell line [55,56]. In agreement with a machine learning approach [55], we find strong co-localization patterns between CTCF and DNA binding proteins Znf143 (29%), JunD (40%), and the myc-associated zinc finger protein Maz (48%) (a full list is provided in Additional file 8). The occupancy of CTCF binding sites again scales with TAD border strength as defined in *Drosophila* (Figure 5e), suggesting that a gradient of combinatorial binding by architectural proteins scales with topological structure and regulatory potential in human cells as well.

In addition to mapping hundreds of distinct factors, the human Encyclopedia of DNA Elements (ENCODE) project has mapped CTCF across dozens of human cell lines and diverse tissues [57], providing a powerful advantage for analyzing cell type-specific versus tissue-invariant CTCF binding sites. We therefore compared CTCF cell type specificity and TAD border localization patterns by analyzing CTCF binding profiles reported across 31 cell lines (62 biological replicates; Figure S6b in Additional file 7). Whereas cell type-specific CTCF binding sites show relatively no enrichment at TAD borders, a striking trend toward TAD border localization is observed with increasing ubiquity, wherein ubiquitous CTCF sites present in all cell lines and biological replicates are most significantly enriched at TAD borders (Figure 5f). These results support Hi-C data proposing that a majority of topological domains are conserved among cell types and even species [3], and further suggest that this tissue-invariant structure may be determined by the constitutive genomic landscape of architectural proteins.

## Discussion

Insulators have been described as regulatory elements capable of activating and repressing transcription [18], able to block enhancer-promoter interactions and, more recently, to facilitate enhancer-promoter communication [58], yet multiple studies in *Drosophila* suggest that many APBSs are not capable of insulator activity at all [13,15]. Architectural proteins are enriched at the borders of TADs [1-3], but why a majority of APBSs localize within topological domains and what differentiates border-associated from non-border binding sites have remained important and unresolved questions. By characterizing and mapping the genome-wide binding profiles for several architectural proteins, including the B box binding subunit of TFIIIC in *D. melanogaster*, we uncover a widespread spectrum of combinatorial binding by architectural proteins that offers an explanation for the diversity of localization patterns and function.

We find that clustering of architectural proteins scales with the tissue-invariant topological domain structure recently described by high-throughput chromosome conformation capture studies. High occupancy APBSs are strongly enriched at TAD borders, and the number of architectural proteins present at a TAD border directly correlates with its strength, as measured by the ratio of inter-TAD versus intra-TAD interaction frequencies. TAD border-associated APBSs represent highly accessible DHS regions present throughout *Drosophila* embryonic development, suggesting the binding of architectural proteins at these sites is constitutive across diverse cell types and may underlie the conserved topological domain structure between different tissues. In support of this conclusion, comparison of CTCF ChIP-seq data across 31 human cell lines suggests that whereas cell type-specific APBSs are virtually unrelated to domain structure, ubiquitous CTCF binding strongly predicts TAD boundary localization.

The clustering of architectural proteins is reminiscent but distinct from the clustering of transcription factors at highly occupied *cis*-regulatory modules, similar to *Drosophila* HOT regions, recently shown to form around the cohesin complex [39,40]. Though CTCF co-occurs with cohesin at a majority of binding sites, it does not localize to cohesin sites associated with mediator and dozens of other transcription factors in humans [39]. Nevertheless, Rad21 is necessary for stabilizing dense transcription factor clusters [40], suggesting the cohesin complex may serve an analogous role at clustered APBSs. Our finding that *Drosophila* architectural proteins, including CTCF, associate with Rad21 further suggests that this role may be evolutionarily conserved.

Genome-wide mapping of condensin complexes extends the relationship between APBSs and SMC-containing complexes even further. High occupancy APBSs are significantly enriched for the condensin II complex, most significantly at a subset of sites bound by Chromator and BEAF-32 (Figure 3c). Comparison of condensin II subunits CAP-H2 and CAP-D3 with the genome-wide CTCF profile in mESCs further suggests that this relationship, like that with cohesin, may be a common feature of high occupancy APBSs. Mammalian CTCF was recently shown to interact with the condensin complex, particularly CAP-D3, both *in vitro* and *in vivo*[59], suggesting CTCF may be responsible for recruiting condensin II to these clustered elements. However, whereas RNAi depletion of dCTCF leads to reduced cohesin localization at low, moderate, and high occupancy APBSs, we find no disruption of CAP-H2 localization to high occupancy APBSs (Additional file 2), suggesting additional factors may play a role in the recruitment of condensin II to these regulatory elements.

What role condensin II plays at APBSs will require future exploration, but many intriguing possibilities arise from its regulated activity throughout the cell cycle. For example, though defined for its involvement in chromosome assembly and segregation, condensin II has been shown to promote the formation of chromosome territories and to be tightly regulated during interphase [60], wherein phosphorylated CAP-H2 is targeted by the ubiquitin ligase complex SCF^Slimb^ for ubiquitin-mediated degradation [61]. CAP-H2 accumulates upon Slimb disruption, leading to chromosome reorganization and nuclear envelope defects, suggesting condensin II levels are tightly regulated for appropriate interphase chromatin organization. Meanwhile, *Drosophila* architectural proteins tightly associate with DNA and remain bound during mitosis [62], particularly at sites aligned with multiple factors, suggesting that condensin-bound APBSs may function as chromatin bookmarks for organized compaction and re-establishment of epigenetic regulation throughout the cell cycle.

**Figure 6:**
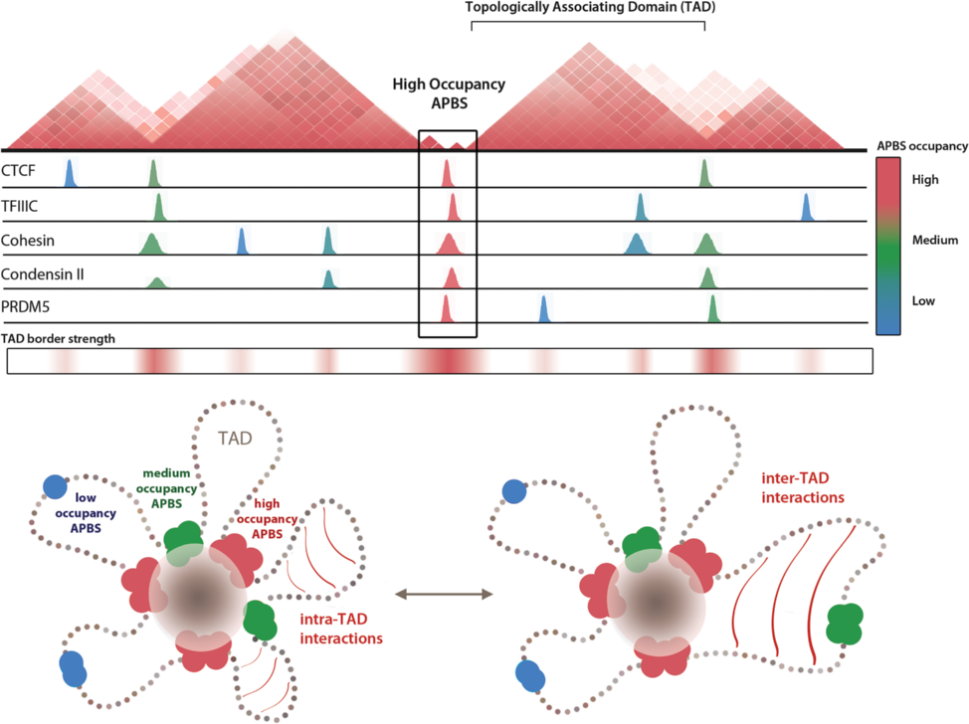
**Combinatorial binding of architectural proteins shapes topological domain structure.** Model illustrating the relationship between protein occupancy at APBSs and observed heterogeneity in TAD border strengths. We uncover a spectrum of architectural protein co-localization, ranging from low (blue) to high (red), which scales with the strength of TAD border formation. We propose that differences in TAD border strength reflect the role of architectural proteins in mediating long-range interactions. Interaction frequencies and/or interaction stability are greatest at high occupancy APBSs (red), whereas fewer or less stable interactions at intermediate APBSs (green) allows for inter-TAD interactions, resulting in comparatively weaker TAD borders observed by Hi-C.

The distinct localization of low versus high occupancy APBSs with respect to TAD borders suggests that function is often context-dependent and modulated by protein composition (Figure 6). Whereas high occupancy APBSs are present at TAD borders and represent genomic loci capable of robust enhancer-blocking activity in transgenic reporter assays, low occupancy APBSs exhibit weak or virtually no enhancer-blocking function, or in the case of Su(Hw), gene repression [13]. These assays are commonly approached using the *gypsy* insulator, composed of 12 clustered Su(Hw) binding sites, as a positive control for such insulator activity, but nevertheless suggest that most APBSs do not represent ‘insulators’ in the classical sense. Instead, low occupancy binding sites localize within TADs and may be reserved for locus-specific gene regulation, such as facilitating enhancer-promoter interactions.

We propose that the spectrum of TAD border strengths accompanied by differences in protein occupancy reflect the role of architectural proteins in long-range interactions (Figure 6) [63,64]. For example, combinatorial binding of architectural proteins and recruitment of SMC-containing cohesin and condensin complexes may increase both their propensity to interact and the stability of interactions with other regulatory elements, strengthened by synergistic protein-protein and protein-DNA interactions. Furthermore, the very nature of high occupancy APBSs may indirectly reflect interactions with proteins bound to discrete genomic loci. In either case, the strong TAD separation defined by clustered APBSs is determined by the likelihood and/or stability of long-range interactions with other regulatory elements. Higher inter-TAD interaction frequencies observed across a comparatively weaker TAD border bound by fewer architectural proteins may be less likely to interact or exhibit weaker, more transient interactions that allow for greater inter-TAD interaction frequencies. A recent study further suggests that APBSs are regulated by poly(ADP-ribosyl)ation of CP190, particularly at low occupancy, independent APBSs [65], whereas high occupancy APBSs more often remain unaffected. The synergy of several factors at clustered APBSs may contribute to this apparent immunity to certain post-translational regulatory mechanisms, which may be directed toward a subset of architectural proteins, and thereby represent a means for establishing stable chromatin domain organization in interphase cells.

## Conclusions

We identify a spectrum of architectural protein occupancy that scales with the topological structure of chromosomes and the regulatory potential of these elements. High occupancy APBSs, which are enriched for both cohesin and condensin complexes, localize to the borders of TADs and represent regions of chromatin that are DNase I hypersensitive throughout *Drosophila* development, suggesting these sites may play a role in establishing the tissue-invariant nature of TADs described in mammals. APBS occupancy scales with the strength of TAD borders, and correlate with the capacity of these elements to function as enhancer-blocking insulators, suggesting the composition of these regulatory elements underlies a spectrum of regulatory potential. We uncover a similar relationship between TFIIIC, CTCF, cohesin, condensin and TADs in mice and humans, suggesting a conserved role for clustered architectural proteins in sub-megabase scale chromatin domain organization.

## Materials and methods

### dTFIIIC220 antibody generation

cDNA corresponding to CG7099 amino acids 1,357 to 1,907 was obtained from the *Drosophila* Genomics Resource Center (DGRC clone LD46862), PCR-amplified introducing a BglII restriction site upstream of the coding sequence, and subcloned into a pET-23a vector containing a glutathione-S-transferase (GST) and His tag at the carboxyl and amino termini, respectively. CG7099 protein fragment expression was induced by IPTG (0.5 mM) in BL21-CodonPlus^®^ Competent Cells grown to a culture density of approximately OD_600_ 0.5, and shaken for approximately 100 rpm for 2 h. Cells were subsequently pelleted and proteins extracted via the B-PER protein extraction reagent (ThermoScientific product number 78243; Waltham, Massachusetts, USA). Polyclonal rabbit antibodies were generated against the isolated CG7099 fragment at the Pocono Rabbit Farm and Laboratory (Canadensis, Pennsylvania, USA). Quality control and antigen specificity were tested by peptide competition assays against Kc167 lysate with rabbit polyclonal α-dTFIIIC220 antibody pre-incubated with bacterial extract expressing GST empty construct or GST-CG7099 construct expressing a fragment corresponding to amino acids 1,357 to 1,907 (Additional file 1).

### Immunoprecipitation and western blot analysis

All steps were performed at 4°C. Kc167 cells were harvested and washed once with ice-cold phosphate-buffered saline (PBS). Cells (0.1 g) were lysed by incubating for 10 minutes with 1 ml of ice-cold PBSMT (2.5 mM MgCl_2_, 3 mM KCl, and 0.3% Triton X-100 in PBS) plus protease inhibitors (1 mM PMSF and Complete protease inhibitor tablet cocktail (Roche, Penzberg, Upper Bavaria, Germany). Lysates were clarified by centrifugation at 16,000 g for 10 minutes and protein concentrations were determined by Bradford assays (Bio-Rad, Hercules, California, USA). Packed Protein A Sepharose (15 μl bead volume; GE Healthcare, Little Chalfont, United Kingdom) was washed three times in PBSMT and pre-incubated with 3 μl of rabbit polyclonal anti-dTFIIIC220 or pre-immune serum for 1 h. Lysate was added to the antibody-conjugated Protein A Sepharose and incubated with agitation for 1 h. Beads were washed three times with 1 ml PBSMT and once with 1 ml PBS. For comparing interaction between dTFIIIC220 and other insulator proteins, 50 μl of 1 M MgCl_2_ was added to the beads and incubated for 5 minutes. Supernatant containing the eluted proteins was isolated by centrifugation. Laemmli SDS buffer was then added to the eluted proteins and boiled for 5 minutes. Samples were resolved by 6% SDS-PAGE and transferred to PVDF membrane (Millipore, Billerica, Massachusetts, USA) in Tris-glycine transfer buffer and 20% methanol for 2 h at 100 volts.

For western blotting, membranes were blocked in TBST (20 mM Tris, pH7.4, 150 mM NaCl, 0.05% Tween 20) with 5% nonfat milk powder and incubated overnight with the following antibodies: rabbit-anti-dTFIIIC220 (1:2,000), rabbit-anti-CP190 (1:10,000), rabbit-anti-Su(Hw) (1:3,000), rabbit-anti-Mod(mdg4)2.2 (1:3,000), guinea pig-anti-dCTCF (1:1,000), mouse-anti-BEAF-32 (1:100; DSHB, Iowa City, Iowa, USA), rabbit-anti-Rad21 (1:1,000; gift from Dr Dale Dorsett) and rabbit-anti-histone H3 (1:3,000; Abcam, Cambridge, United Kingdom). Membranes were washed three times with TBST and probed with secondary antibodies-conjugated to horse radish peroxidase (1:5,000; Jackson ImmunoResearch Laboratories, West Grove, Pennsylvania, USA) for 1 h. After three more washes, the presence of different proteins was detected using SuperSignal West Pico/Dura Chemiluminescent substrate (Thermo Scientific, Waltham, Massachusetts, USA).

### ChIP-seq and reference data

ChIP was performed as previously described [66]. In addition to dTFIIIC220, ChIP for Rad21, Barren, and CAP-H2 in *Drosophila* Kc cells was carried out using previously described antibodies (Rad21, α-Rabbit [41]; Barren, α-Rabbit [67]; CAP-H2, α-Rabbit; gifts from Dr Dale Dorsett, Dr Hugo Bellen, and Dr Giovanni Bosco, respectively). Sequences were mapped to the dm3 genome with Bowtie 0.12.3 [68] using default settings. To account for the repetitive nature of tRNA genes, multimapping sequences were filtered out for all dTFIIIC220 ChIP-seq experiments. Peaks were then called with MACS 1.4.0alpha2 [30] using equal numbers of unique reads for input and ChIP samples, a *P* value cutoff of 1 × 10^-10^, and a false discovery rate threshold of 5% (Additional file 9). For classification of overlapping APBSs, MACS-identified peaks (pval 1e-10, false discovery rate 5%) are further refined as the MACS calculated summit ±200 bp. For dTFIIIC220, peaks used for analyses were independently identified by MACS in two out of three biological replicates. For visualization, mapped sequence reads were loaded on to the Integrated Genomics Viewer [69,70]. Previously published ChIP-seq data for *Drosophila* architectural proteins were obtained from Gene Expression Omnibus (GEO) accessions GSE30740 [27] and GSE36944 [15], and ChIP-chip data corresponding to TFIIIB subunits TRF1 and BRF from [71]. Raw DNase-seq in Kc167 cells was obtained from [51]; DNase-seq in HeLa S3 cells from GEO series GSE32970; and DNase-seq in mESCs from ENCODE dataset wgEncodeUwDgfEscj7129 [57]. ChIP-seq data for architectural proteins in mESCs were obtained from GEO series GSE29218 (CTCF), GSE33346 (Rad21, CAP-H2, CAP-D3) [42], and GSE51816 (PRDM5), and from ArrayExpress accession E-MTAB-767 (TFIIIC-110 -220 -90). ChIP-seq data for architectural proteins in HeLa S3 and K562 cells were obtained from GEO series GSE31477 (TFIIIC, Rad21) and from publicly available ENCODE data [55,56,72].

### Bioinformatics analyses

Sequence alignment for the dTFIIIC220 B box binding domain with analogous proteins in yeast and humans was generated using the Conserved Domains Database [73] and visualized using C3nD v4.3 [74-76] and Jalview [77]. For ChIP-seq comparisons, DNA sequence motifs were identified by MEME-ChIP using default settings [28]. Overlap between dTFIIIC220 and annotated tRNA genes were identified using publicly available tools on Galaxy [78-80]. Comparison of APBSs with respect to Pol II-transcribed genes employed gene structure (transcription start sites, exons, introns, transcription termination sites) obtained using the UCSC genome browser [81,82]. Enrichment profiles for architectural protein co-occurrence and localization to TAD boundaries were defined as the observed overlapping frequencies over expected frequencies determined by shuffling datasets, while controlling for the number of peaks and start/stop location of peaks on each chromosome. *P* values were determined as the chance of observing an equal or greater co-occurrence across 100,000 Monte Carlo permutation tests. Results were visualized using Java Treeview [83]. Unless otherwise noted, read intensity plots were generated by binning ChIP-seq reads into 100 bp bins and extracting read counts in bins surrounding described anchor points (for example, dTFIIIC220 summits), and visualized using Java Treeview [83]. Rank-order normalization of DNase-seq and/or ChIP-seq data was carried out as recently described [84]. Briefly, datasets are rank-ordered in 10-bp bins across the reference genome, descending from high to low read intensity, and at each level, bins are re-assigned the average read value across samples used for comparison.

### Overlap matrices and classification of APBSs

#### D. melanogaster

ChIP-seq peaks, defined as 400 bp centered around MACS calculated summits, were cross-analyzed using BED tools MultiIntersectBed [85], creating a matrix of unique genomic loci bound by architectural proteins. In *Drosophila* this includes ChIP-seq data for dCTCF, BEAF-32, Su(Hw), CP190, Mod(mdg4), DREF, Chromator, L(3)mbt, dTFIIIC220, Rad21, and CAP-H2. The number of target motifs and the relative level of ChIP tag density were not considered when generating this list. Adjacent output peaks were merged and the largest occupancy region and associated factors isolated for further analyses (that is, directly adjacent regions bound by four, five, then four proteins were merged into one peak centered on the highest (five proteins) occupied region; Additional files 3 and 7). Each APBS was then classified as being either low occupancy (one to three proteins), moderate occupancy (four to six proteins), or high occupancy (seven or more proteins). Co-localization frequencies for factors depicted in Figure 3c were calculated similarly and correspond to sites combinatorially bound by four or more architectural proteins identified independently of dTFIIIC220, cohesin, and condensin I or II.

### Mouse embryonic stem cells

ChIP-seq peak data for CTCF (GSE 29218), cohesin (GSE 33346), TFIIIC, condensin (GSE 33346), and PRDM5 (GSE 51816) in mESCs were obtained from published sources. ChIP-seq experiments for multiple subunits were available for TFIIIC (-220, -110, and -90) and condensin (CAP-H2 and CAP-D3). For these cases data from all available subunits were combined into a single set. Being in proximity to any subunit of TFIIIC or condensin was considered sufficient for co-localization. CTCF sites were classified as in proximity to Rad21 or TFIIIC if there was a Rad21 or TFIIIC peak within 500 bases of the center of the CTCF site. As most CTCF sites had Rad21, the number of CTCF sites with TFIIIC and without Rad21 was very small and therefore is not shown. Each of these unique subsets was then assayed for its prevalence near TAD borders. Sites within 20 kb of a border were considered at a TAD border and sites outside of these windows were considered not at a TAD border. Expected values were calculated using a random distribution of sites with site type, size, and chromosomes conserved and locations randomized. We performed a Monte Carlo permutation test in order to calculate significance. The classifications of the sites were randomized and the number of permutations that resulted in a result as extreme as the observed over the total number of permutations was taken as the *P* value.

### Humans

Occupancy at CTCF binding sites were determined by cross-comparison with publicly available genomewide binding datasets for DNA-binding/transcription factors CTCF, Rad21, TF3C, Yy1, Smc3, Znf143, Myc, Max, Maz, JunD, Arid3a, Atf1, Atf3, Bach1, Bcl, Bcl3, Bdp1, Bhlhe40, Brf1, Brf2, Brg1, Cbx3, CCNT2, CEBPbeta, CHD2, Corest, CTCFL, E2F4, E2F6, Egr1, Elf1, Elk1, Ets1, Ezh2, fos, FosL, GATA1, GATA2, HDAC1, HDAC2, HDAC6, HDAC8, HMGN3, Ini1, cJun, MafF, MafK, Mef2a, MXI1, Nelfe, Nfe2, Nfya, Nfyb, Nr2f2, Nrf1, P300, Phf8, Plu1, Rbbp5, Rfx5, Sap30, Setdb1, Sin3A, Sirt6, Six5, Sp1, Sp2, Srf, Stat5, Taf1, Taf7, Tal1, Tblr1, Tbp, Tead4, TFIIB, TFIIF, Thap1, Tr4, Trim28, Ubtfs, Usf1, Usf2, Xrcc4, Zbtb7, Zbtb33, Znf263, and Znf274 [22,56,57]. Overlap matrices were generated as described for *Drosophila*.

#### Topologically associating domains and calculation of their border strength

Hi-C analysis and definition of TADs in *Drosophila* Kc167 cells were used as previously reported [1]. To measure the degree of separation of chromatin between two sides of a specific enzyme cutting site S, we analyze region A, which is adjacent to S on one side, and region B flanking cut site S on the opposite side. Intra-TAD Hi-C interaction counts within A and intra-TAD Hi-C interaction counts within B are calculated and compared with inter-TAD Hi-C interaction counts between regions A and B. The difference is defined as local contrast and centered to have median value of approximately 1. High value of local contrast corresponds to enriched intra-domain contact frequencies relative to inter-domain contacts. Thus, TAD borders generally exhibit strong measures of local contrast. TADs defined in mESCs and humans were obtained from published data [3]. TAD borders were taken from hESC and IMR90 lines and a common subset of borders found in both was used to form the conserved dataset. TAD border strengths in mESCs and humans were calculated as described for *Drosophila.*

#### Comparison of APBS occupancy and insulator function from transgenic reporter assays

Enhancer-blocking results reported for several tested insulator elements were obtained from work by Nègre *et al*. [52] and Schwartz *et al*. [13], and categorized as either capable of robust enhancer blocking, weak/context dependent enhancer blocking, no enhancer blocking, or in the case of Schwartz *et al*., two suppressor of hairy wing independent loci capable of gene repression. The occupancy of each insulator element was then extracted by comparison with ChIP-seq peaks and overlap matrices (Additional file 10).

#### CTCF site ubiquity

Existing CTCF ChIP-seq data were obtained from the ENCODE project for analysis. Thirty-one cell lines with two replicates each were chosen for a total of 62 unique ChIP-seq experiments in a wide range of human cell lines (Additional file 11). These 62 data sets were combined into a composite list of all CTCF sites classified by the number of experiments each was found in. Sites that were found in only 1 of the 62 experiments were discarded as they failed to replicate. Sites less than a thousand bases from a site present in over twice as many cell lines were merged into the more ubiquitous site. To create an expected distribution, CTCF sites were shuffled. The ubiquity, size, and chromosome of each site were conserved, but the locations were randomized to a position between the first and the last CTCF sites on the chromosome. Sites were then separated into eight bins of approximately 15,000 sites by their ubiquity. The ubiquity scores of each bin and number of CTCF sites are as follows: bin 1, 2 replicates, 15,568 sites; bin 2, 3 to 4 replicates, 14,328 sites; bin 3, 5 to 8 replicates, 14,326 sites; bin 4, 9 to 17 replicates, 15,240 sites; bin 5, 18 to 33 replicates, 14,536 sites; bin 6, 34 to 52 replicates, 15,707 sites; bin 7, 53 to 61 replicates, 15,213 sites; bin 8, 62 replicates, 15,582 sites. To analyze localization to hESC TAD borders, each site in the observed and expected data sets was classified as within 20 kb of a TAD border or not. The resulting frequencies were used to calculate observed over expected values.

### Human cell lines and corresponding GEO accession numbers

#### CTCF, cohesin, and TFIIIC analysis in HeLa S3 cells

Enrichment of CTCF, TFIIIC, and Rad21 at human TAD borders (Additional file 7) was performed using CTCF, TFIIIC, and Rad21 datasets commonly mapped in HeLa S3 cells. Published ChIP-exo experiments were used as HeLa CTCF sites without any additional modification [72]. Rad21 and TFIIIC sites were determined from previously published ChIP-seq experiments (GSE31477). TAD borders were taken from hESC and IMR90 lines and a common subset of borders found in both was used to form the conserved dataset. CTCF sites were classified as in proximity to Rad21 or TFIIIC if there was a Rad21 or TFIIIC peak within 500 bases of the center of the CTCF site. As most CTCF sites had Rad21, the number of CTCF sites with TFIIIC and without Rad21 was very small and therefore is not shown. Each of these unique subsets was then assayed for its prevalence near TAD borders. Sites within 20 kb of a border were considered at a TAD border and sites outside of these windows were considered not at a TAD border. Expected values were calculated using a random distribution of sites with site type, size, and chromosomes conserved and locations randomized. We performed a Monte Carlo permutation test in order to calculate significance. The classifications of the sites were randomized and the number of permutations that resulted in a result as extreme as the observed over the total number of permutations was taken as the *P* value.

### Accession numbers

All ChIP-seq data are publicly available under GEO accession number GSE54529.

## Abbreviations

APBS: architectural protein binding site; bp: base pair; ChIP: chromatin immunoprecipitation; DHS: DNase I hypersensitivity; ESC: embryonic stem cell; GEO: Gene Expression Omnibus; GST: glutathione-S-transferase; hESC: human embryonic stem cell; mESC: mouse embryonic stem cell; PBS: phosphate-buffered saline; Pol: RNA polymerase; RNAi: RNA interference; TAD: topologically associating domain; tDNA: tRNA gene.

## Competing interests

The authors declare that they have no competing interests.

## Authors’ contributions

KVB and VGC conceived the project. KVB, CTO, and NT performed molecular experiments, and generated and characterized dTFIIIC220 antibodies. KVB, MHN, and LL carried out bioinformatic analyses. LL and ZSQ conceived and designed TAD boundary strength calculation. KVB and VGC drafted the manuscript. All authors read and approved the final manuscript.

## Supplementary Material

Additional file 1: Figure S1 *CG7099* is the predicted *Drosophila* TFIIIC B-box binding subunit (related to Figure 1). **(a,b)** Gene structure and sequence alignment for the B box binding domain of CG7099, predicted by the Conserved Domain Database and generated using Cn3D v4.3, with TFIIIC B box binding subunits in *D. melanogaster, Drosophila simulans, Drosophila pseudoobscura, Drosophila virilis, S. cerevisiae*, *S. pombe*, *Mus musculus*, and *Homo sapiens* (gi74709141 - HsTFIIIC220). **(c)** Percentage sequence identity for dTFIIIC220 with homologous proteins in yeast and mammals, with respect to the full protein (black) and predicted B box binding domain (pink). **(d)** Generation of a dTFIIIC220 specific antibody; immunoblot staining against Kc167 lysate with pre-immune versus rabbit polyclonal α-dTFIIIC220 antibody. dTFIIIC220 migrates at the predicted molecular weight of 220 kDa. **(e)** Peptide competition assay: immunoblot staining against Kc167 lysate with rabbit polyclonal α-dTFIIIC220 antibody pre-incubated with bacterial extract expressing GST empty construct (left) or GST-CG7099 construct expressing a fragment corresponding to amino acids 1,357 to 1,907. **(f)** Immunofluorescence localization of dTFIIIC220 on *Drosophila* polytene chromosomes (green) reveals staining at discrete bands and nucleolar structures, as evidence by co-staining against the ribonucleoprotein fibrillarin (red). Click here for file

Additional file 2: Figure S2 Relationship between SMC-containing cohesin and condensin complexes and *Drosophila* architectural proteins (related to Figures 2 and 3). **(a)** Average Rad21 ChIP-seq tag density at sites bound near transcription start sites (promoter) versus enhancers marked by H3K4me1 and H3K27ac reveals higher occupancy at enhancers as previously described [41]. Rad21 ChIP-seq tag density at APBSs versus non-APBSs shows higher occupancy at sites co-bound by either dCTCF, BEAF-32, or Su(Hw). **(b)** Example genomics viewers illustrating overlapping peaks for Rad21, Barren, and/or CAP-H2 at sites bound by architectural proteins dTFIIIC220, dCTCF, BEAF-32, or Su(Hw). **(c)** Rank-order normalized tag densities comparing Rad21, Barren, and CAP-H2 enrichment at sites bound by architectural proteins dTFIIIC220, dCTCF, BEAF-32, or Su(Hw). **(d)** Percentage of APBSs overlapping Rad21, Barren, or CAP-H2 for dTFIIIC220, dCTCF, BEAF-32, or Su(Hw). **(e)** Heatmap representation of Rad21 peaks affected by RNAi depletion of architectural protein dCTCF (chromatin preparation and dCTCF knockdown levels previously published [15]; Additional file 9): 744 sites are reduced >67% (top), whereas approximately 5,300 sites remain comparatively unaffected (bottom). **(f)** Rad21 sites affected by dCTCF RNAi correspond to sites where Rad21 overlaps dCTCF. Median fold change in Rad21 signal at sites co-bound by dCTCF versus independent of dCTCF (top). Average profile of dCTCF at Rad21 peaks affected by dCTCF RNAi (bottom: dotted line) versus sites unaffected (bottom: solid line). **(g)** Median fold change in ChIP-seq signals for dTFIIIC220, CP190, Rad21, and CAP-H2 in response to dCTCF RNAi; divided into sites defined as high occupancy (red), intermediate occupancy (green), and low occupancy (blue) APBSs. Click here for file

Additional file 3: Table S1 A list of all architectural protein binding sites (APBSs) in Kc167 cells determined by ChIP-seq, with location, occupancy level, and matrix of factors present. Click here for file

Additional file 4: Figure S3 Architectural protein binding site (APBS) occupancy and relation to genome organization (related to Figure 4). **(a)** APBSs defined by occupancy of architectural proteins dTFIIIC220, dCTCF, BEAF-32, Su(Hw), CP190, Mod(mdg4), DREF, Chromator, L(3)mbt, and SMC complex proteins Rad21 and CAP-H2. Example Genomics viewer illustrating high occupancy APBSs bound by all criteria. APBS occupancy was categorized into groups of low (1 to 3), medium (4 to 6), or high (7 to 11) occupancy for subsequent analyses. Size distribution of APBSs (bp) centered on genomic fragments of highest occupancy. **(b)** Average DNase-seq tag density over APBSs, at each stage of protein occupancy. **(c)** Average distance profile (top) and DNase activity (bottom) of APBSs with respect to gene structure (transcription start site), as a function of occupancy. **(d)** Percentage of APBSs within 5-kb bins of TAD borders defined in Kc167 cells [1]. Less than 20% of mapped high occupancy APBSs are greater than 30 kb from TAD borders. **(e)** Fraction of APBSs within 1-kb bins of TAD borders defined in *Drosophila* embryos (top) [2], and comparison with non-promoter APBSs (bottom). **(f)** Comparison of APBS occupancy and TADs defined in *Drosophila* embryos (related to Figure 4b): 48% of embryonic TAD borders are delineated by a high occupancy APBS, 35% by medium occupancy APBSs, and 12% by low occupancy APBSs, plus or minus 2 kb (4-kb window total); 4% of TAD borders do not correlate with any APBSs (TAD borders n = 1,169, high occupancy APBSs n =1,638, *P* < 0.00001 permutation test). Click here for file

Additional file 5: Figure S4 APBS occupancy is maintained throughout *Drosophila* development (related to Figure 4). **(a)** Heatmap representation of ChIP-seq tag densities for dCTCF, BEAF-32, Su(Hw), CP190, Chromator, and Cohesin (Rad21 or SMC3) at high, medium, and low occupancy APBSs in Kc167 cells (left) or late embryonic (for SMC3, larvae third instar) samples. **(b)** Tag density plots of rank-order normalized DNase-seq profiles throughout embryonic stages of development at all APBSs. **(c)** Tag density plots of rank-order normalized DNase-seq profiles throughout embryonic stages of development at APBSs that are not associated with gene promoters. Click here for file

Additional file 6: Figure S5 Relationship between APBS density and topological structure. **(a)** Visualization of pairwise interaction frequencies on an megabse scale illustrates the enrichment of high occupancy APBSs at TAD borders (white lines), and the heterogeneity of TAD size across the genome. **(b)** The occupancy of APBSs negatively correlates with the size of local TAD structure. **(c)** The density of APBSs within 10 kb is inversely correlated with local TAD size. Click here for file

Additional file 8: Table S2 A list of all CTCF binding sites determined by ChIP-seq in human K562 cells, with location, occupancy level, and matrix of factors present based on publicly available ENCODE data. Click here for file

Additional file 7: Figure S6 Characterization of human CTCF binding sites (related to Figure 5). **(a)** Enrichment of human CTCF binding sites with respect to TAD borders defined in IMR90 fibroblasts (red), human embryonic stem cells (blue), or TAD borders conserved between these cell lines (black), when bound alone, with Rad21, or with both Rad21 and TFIIIC. **(b)** Histogram of CTCF binding sites with respect to cell-type specificity. CTCF peaks across 31 human cell lines obtained from the Encyclopedia of DNA Elements (ENCODE) [57] ordered by the number of experiments (biological replicates). Approximately 15,000 CTCF binding sites are independently identified in all 31 cell lines and 62 biological replicates. Click here for file

Additional file 9: Figure S7 ChIP-seq threshold statistics and relationship between MACS *P* values and false discovery rates (FDRs). Vertical and horizontal dashed (red) lines represent *P* value and FDR cutoff statistics used to determine ChIP-peak profiles for dTFIIIC220, Rad21, CAP-H2, and Barren (grey box). Click here for file

Additional file 10: Table S3 A list of all tested insulator sequences and enhancer-blocking outcomes in transgenic reporter assays determined by Nègre *et al*. [52] and Schwartz *et al*. [13], and categorized as either capable of robust enhancer blocking, weak/context dependent enhancer blocking, no enhancer blocking, or gene repression. Occupancy of each insulator element extracted from ChIP-seq data and overlap matrices. Click here for file

Additional file 11: Table S4 A list of all 31 human cell lines and GEO accession numbers for CTCF binding data obtained from ENCODE datasets. CTCF ChIP-seq data were selected as those with biological replicates (62 datasets). Click here for file
