# Supplementary figures and images for: Insulator function and topological domain border strength scale with architectural protein occupancy
Source: Genome Biol. 2014 Jun 30;15(5):R82. doi: 10.1186/gb-2014-15-5-r82 (PMC4226948; doi:10.1186/gb-2014-15-5-r82)

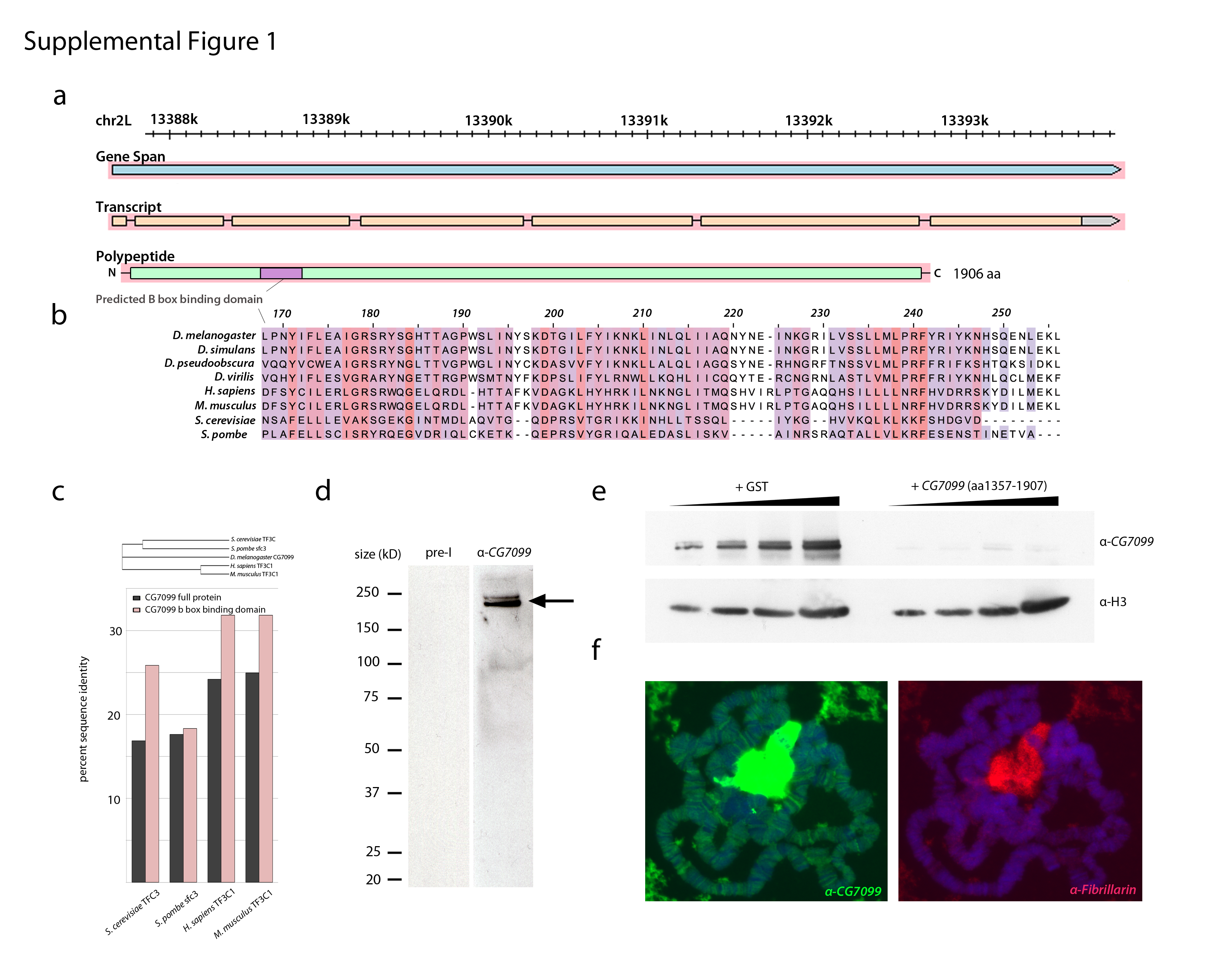

Supplement: Additional file 1: Figure S1 — CG7099 is the predicted Drosophila TFIIIC B-box binding subunit (related to Figure 1). (a,b) Gene structure and sequence alignment for the B box binding domain of CG7099, predicted by the Conserved Domain Database and generated using Cn3D v4.3, with TFIIIC B box binding subunits in D. melanogaster, Drosophila simulans, Drosophila pseudoobscura, Drosophila virilis, S. cerevisiae, S. pombe, Mus musculus, and Homo sapiens (gi74709141 - HsTFIIIC220). (c) Percentage sequence identity for dTFIIIC220 with homologous proteins in yeast and mammals, with respect to the full protein (black) and predicted B box binding domain (pink). (d) Generation of a dTFIIIC220 specific antibody; immunoblot staining against Kc167 lysate with pre-immune versus rabbit polyclonal α-dTFIIIC220 antibody. dTFIIIC220 migrates at the predicted molecular weight of 220 kDa. (e) Peptide competition assay: immunoblot staining against Kc167 lysate with rabbit polyclonal α-dTFIIIC220 antibody pre-incubated with bacterial extract expressing GST empty construct (left) or GST-CG7099 construct expressing a fragment corresponding to amino acids 1,357 to 1,907. (f) Immunofluorescence localization of dTFIIIC220 on Drosophila polytene chromosomes (green) reveals staining at discrete bands and nucleolar structures, as evidence by co-staining against the ribonucleoprotein fibrillarin (red). [file gb-2014-15-5-r82-S1.tiff]

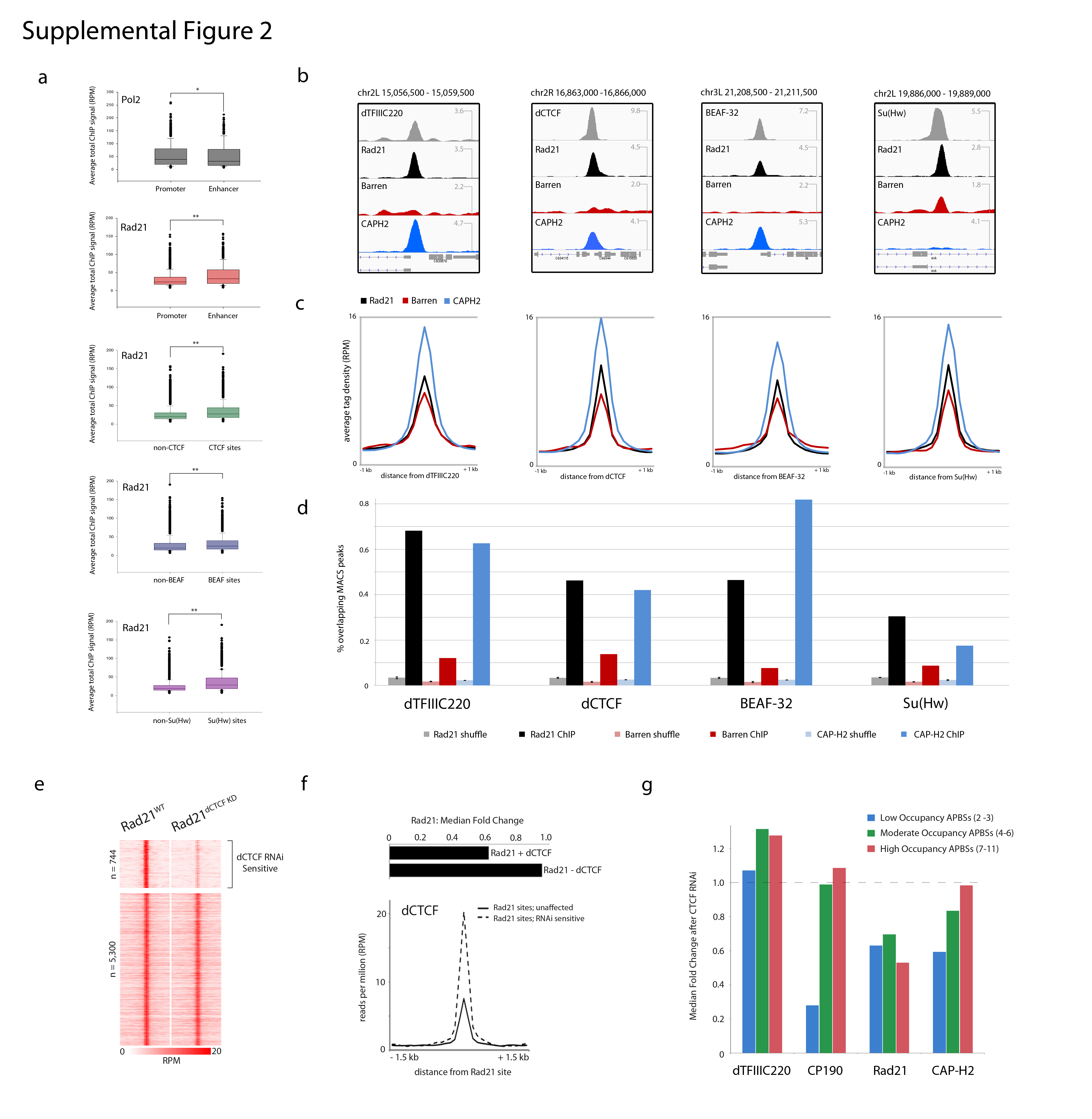

Supplement: Additional file 2: Figure S2 — Relationship between SMC-containing cohesin and condensin complexes and Drosophila architectural proteins (related to Figures 2 and 3). (a) Average Rad21 ChIP-seq tag density at sites bound near transcription start sites (promoter) versus enhancers marked by H3K4me1 and H3K27ac reveals higher occupancy at enhancers as previously described [41]. Rad21 ChIP-seq tag density at APBSs versus non-APBSs shows higher occupancy at sites co-bound by either dCTCF, BEAF-32, or Su(Hw). (b) Example genomics viewers illustrating overlapping peaks for Rad21, Barren, and/or CAP-H2 at sites bound by architectural proteins dTFIIIC220, dCTCF, BEAF-32, or Su(Hw). (c) Rank-order normalized tag densities comparing Rad21, Barren, and CAP-H2 enrichment at sites bound by architectural proteins dTFIIIC220, dCTCF, BEAF-32, or Su(Hw). (d) Percentage of APBSs overlapping Rad21, Barren, or CAP-H2 for dTFIIIC220, dCTCF, BEAF-32, or Su(Hw). (e) Heatmap representation of Rad21 peaks affected by RNAi depletion of architectural protein dCTCF (chromatin preparation and dCTCF knockdown levels previously published [15]; Additional file 9): 744 sites are reduced >67% (top), whereas approximately 5,300 sites remain comparatively unaffected (bottom). (f) Rad21 sites affected by dCTCF RNAi correspond to sites where Rad21 overlaps dCTCF. Median fold change in Rad21 signal at sites co-bound by dCTCF versus independent of dCTCF (top). Average profile of dCTCF at Rad21 peaks affected by dCTCF RNAi (bottom: dotted line) versus sites unaffected (bottom: solid line). (g) Median fold change in ChIP-seq signals for dTFIIIC220, CP190, Rad21, and CAP-H2 in response to dCTCF RNAi; divided into sites defined as high occupancy (red), intermediate occupancy (green), and low occupancy (blue) APBSs. [file gb-2014-15-5-r82-S2.tiff]

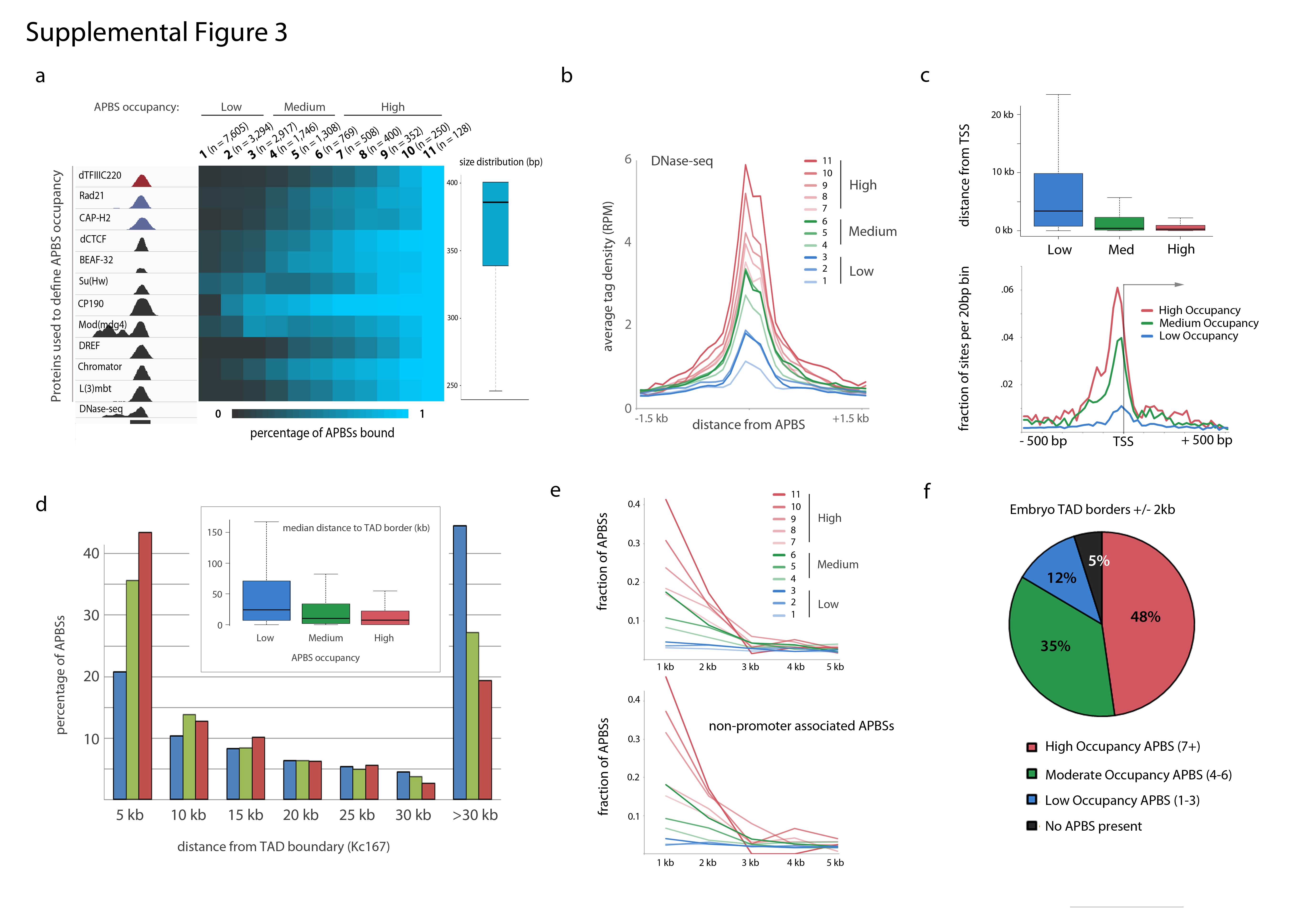

Supplement: Additional file 4: Figure S3 — Architectural protein binding site (APBS) occupancy and relation to genome organization (related to Figure 4). (a) APBSs defined by occupancy of architectural proteins dTFIIIC220, dCTCF, BEAF-32, Su(Hw), CP190, Mod(mdg4), DREF, Chromator, L(3)mbt, and SMC complex proteins Rad21 and CAP-H2. Example Genomics viewer illustrating high occupancy APBSs bound by all criteria. APBS occupancy was categorized into groups of low (1 to 3), medium (4 to 6), or high (7 to 11) occupancy for subsequent analyses. Size distribution of APBSs (bp) centered on genomic fragments of highest occupancy. (b) Average DNase-seq tag density over APBSs, at each stage of protein occupancy. (c) Average distance profile (top) and DNase activity (bottom) of APBSs with respect to gene structure (transcription start site), as a function of occupancy. (d) Percentage of APBSs within 5-kb bins of TAD borders defined in Kc167 cells [1]. Less than 20% of mapped high occupancy APBSs are greater than 30 kb from TAD borders. (e) Fraction of APBSs within 1-kb bins of TAD borders defined in Drosophila embryos (top) [2], and comparison with non-promoter APBSs (bottom). (f) Comparison of APBS occupancy and TADs defined in Drosophila embryos (related to Figure 4b): 48% of embryonic TAD borders are delineated by a high occupancy APBS, 35% by medium occupancy APBSs, and 12% by low occupancy APBSs, plus or minus 2 kb (4-kb window total); 4% of TAD borders do not correlate with any APBSs (TAD borders n = 1,169, high occupancy APBSs n =1,638, P < 0.00001 permutation test). [file gb-2014-15-5-r82-S4.tiff]

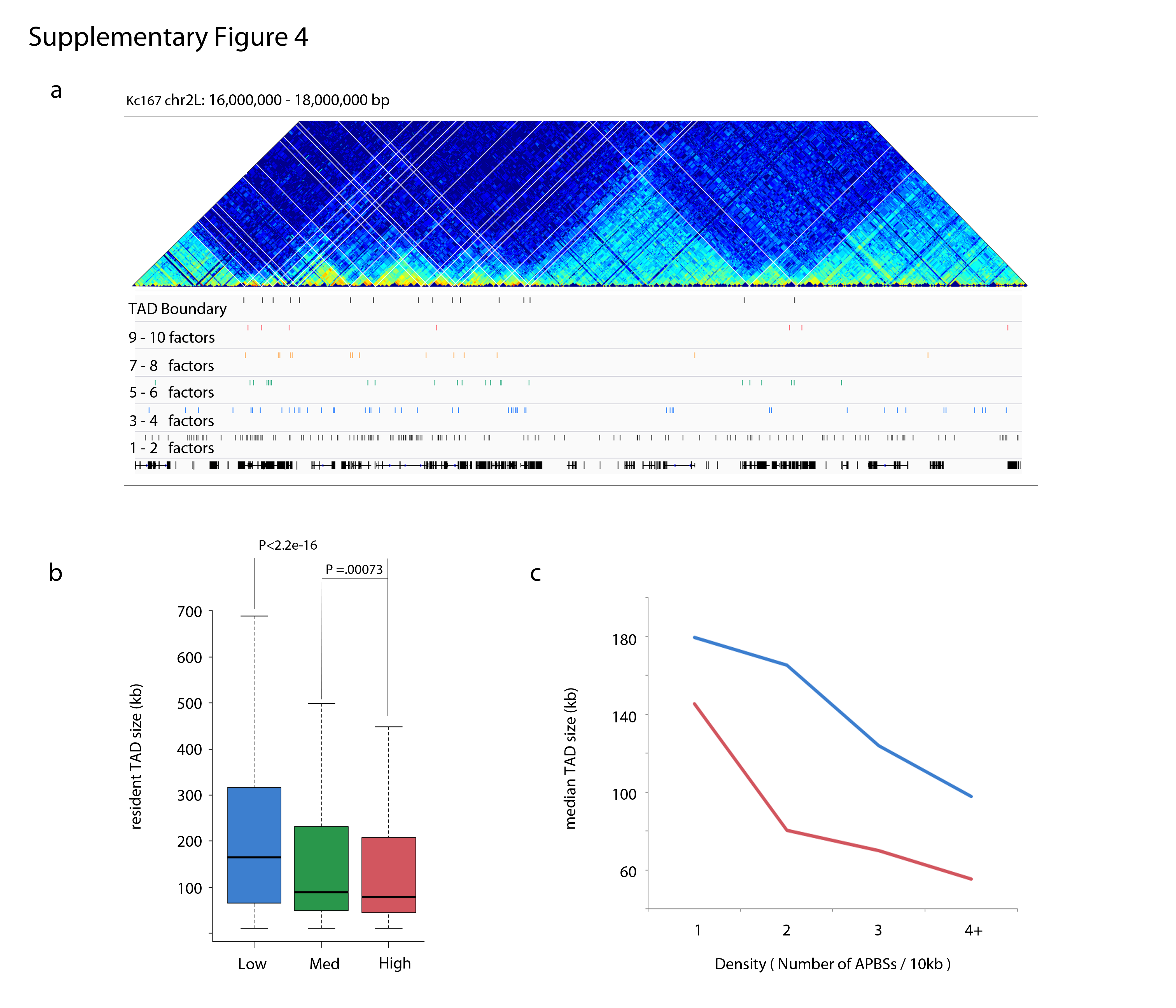

Supplement: Additional file 5: Figure S4 — APBS occupancy is maintained throughout Drosophila development (related to Figure 4). (a) Heatmap representation of ChIP-seq tag densities for dCTCF, BEAF-32, Su(Hw), CP190, Chromator, and Cohesin (Rad21 or SMC3) at high, medium, and low occupancy APBSs in Kc167 cells (left) or late embryonic (for SMC3, larvae third instar) samples. (b) Tag density plots of rank-order normalized DNase-seq profiles throughout embryonic stages of development at all APBSs. (c) Tag density plots of rank-order normalized DNase-seq profiles throughout embryonic stages of development at APBSs that are not associated with gene promoters. [file gb-2014-15-5-r82-S5.tiff]

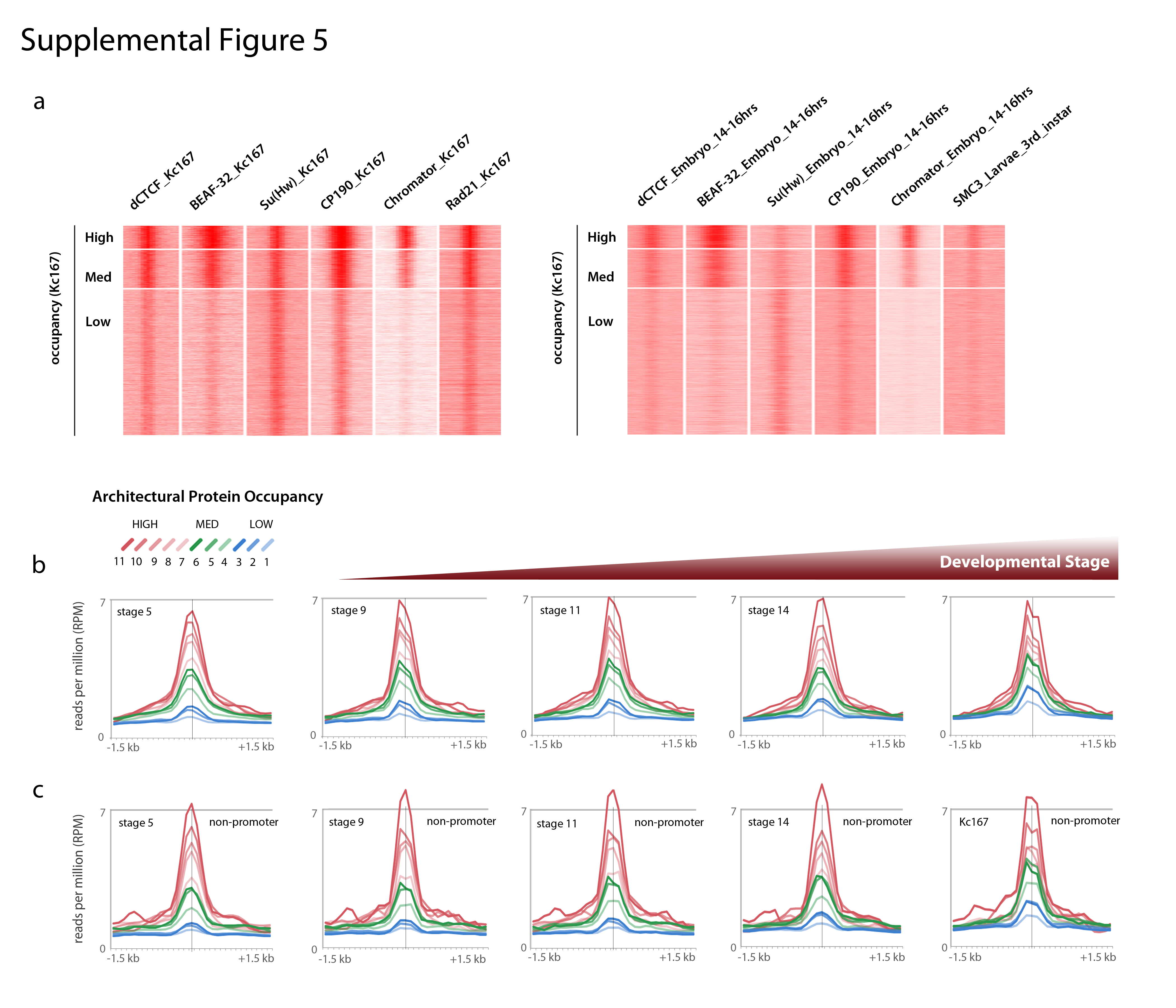

Supplement: Additional file 6: Figure S5 — Relationship between APBS density and topological structure. (a) Visualization of pairwise interaction frequencies on an megabse scale illustrates the enrichment of high occupancy APBSs at TAD borders (white lines), and the heterogeneity of TAD size across the genome. (b) The occupancy of APBSs negatively correlates with the size of local TAD structure. (c) The density of APBSs within 10 kb is inversely correlated with local TAD size. [file gb-2014-15-5-r82-S6.tiff]

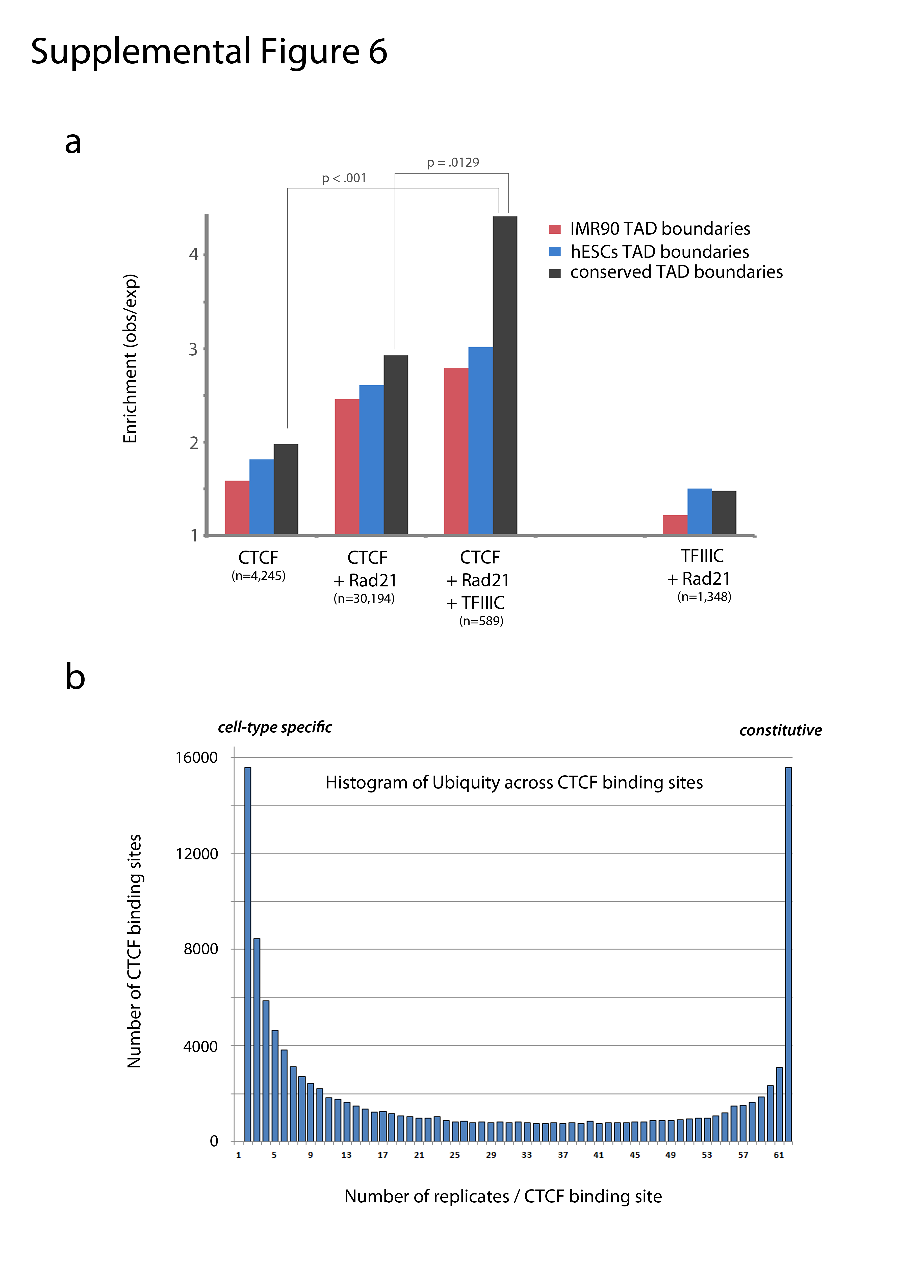

Supplement: Additional file 7: Figure S6 — Characterization of human CTCF binding sites (related to Figure 5). (a) Enrichment of human CTCF binding sites with respect to TAD borders defined in IMR90 fibroblasts (red), human embryonic stem cells (blue), or TAD borders conserved between these cell lines (black), when bound alone, with Rad21, or with both Rad21 and TFIIIC. (b) Histogram of CTCF binding sites with respect to cell-type specificity. CTCF peaks across 31 human cell lines obtained from the Encyclopedia of DNA Elements (ENCODE) [57] ordered by the number of experiments (biological replicates). Approximately 15,000 CTCF binding sites are independently identified in all 31 cell lines and 62 biological replicates. [file gb-2014-15-5-r82-S7.tiff]

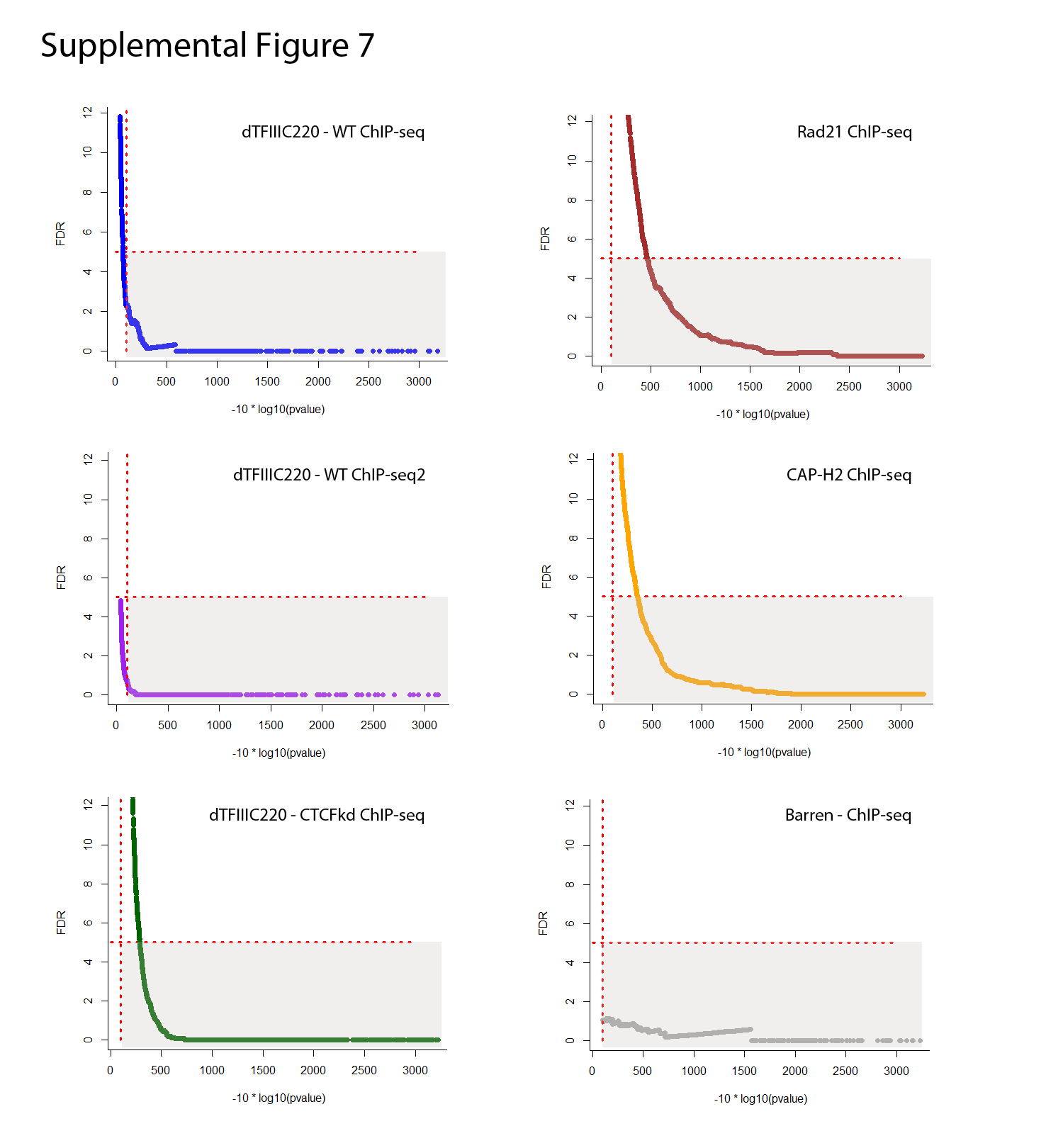

Supplement: Additional file 9: Figure S7 — ChIP-seq threshold statistics and relationship between MACS P values and false discovery rates (FDRs). Vertical and horizontal dashed (red) lines represent P value and FDR cutoff statistics used to determine ChIP-peak profiles for dTFIIIC220, Rad21, CAP-H2, and Barren (grey box). [file gb-2014-15-5-r82-S9.tiff]
